# Population sequencing of cherry accessions unravels the evolution of *Cerasus* species and the selection of genetic characteristics in edible cherries

**DOI:** 10.1186/s43897-024-00120-4

**Published:** 2025-01-08

**Authors:** Yahui Lei, Songtao Jiu, Yan Xu, Baozheng Chen, Xiao Dong, Zhengxin Lv, Anthony Bernard, Xunju Liu, Lei Wang, Li Wang, Jiyuan Wang, Zhuo Zhang, Yuliang Cai, Wei Zheng, Xu Zhang, Fangdong Li, Hongwen Li, Congli Liu, Ming Li, Jing Wang, Jijun Zhu, Lei Peng, Teresa Barreneche, Fei Yu, Shiping Wang, Yang Dong, Dirlewanger Elisabeth, Shengchang Duan, Caixi Zhang

**Affiliations:** 1 Department of Plant Science, School of Agriculture and Biology, Shanghai Jiao Tong University, Shanghai, 200240 P. R. China; 2 College of Science, Yunnan Agricultural University, Kunming, Yunnan 650201 P. R. China; 3 College of Food Science and Technology, Yunnan Agricultural University, Kunming, 650201 Yunnan P. R. China; 4 UMR BFP, INRAE, Univ. Bordeaux, 71 Avenue Edouard Bourlaux, 33882 Villenave d’Ornon, France; 5 College of Horticulture, Northwest A&F University, Yangling, Shaanxi 712100 P. R. China; 6 Dalian Academy of Agricultural Sciences, Dalian, Liaoning 116036 P. R. China; 7 Yantai Academy of Agricultural Sciences, Yantai, Shandong 265500 P. R. China; 8 Horticulture Research Institute, Sichuan Academy of Agricultural Sciences, Sichuan, 610066 P. R. China; 9 Zhengzhou Fruit Tree Research Institute, Chinese Academy of Agricultural Sciences, Zhengzhou, Henan 450009 P. R. China; 10 Forestry and Fruit Research Institute, Beijing Academy of Agriculture and Forestry Sciences, Beijing, P. R. China; 11 Shanghai Botanical Garden, Shanghai, 200231 P. R. China; 12 College of Landscape and Horticulture, Yunnan Agricultural University, Kunming, Yunnan 650201 P. R. China; 13 Horticultural Research Institute, Yunnan Academy of Agricultural Sciences, Kunming, Yunnan 650201 P. R. China; 14 College of Plant Protection, Yunnan Agricultural University, Kunming, Yunnan 650201 P. R. China

**Keywords:** *Cerasus* subgenus, Population structure, Genetic diversity, Edible cherries, Selection signatures

## Abstract

*Cerasus* is a subgenus of *Prunus* in the family Rosaceae that is popular owing to its ornamental, edible, and medicinal properties. Understanding the evolution of the *Cerasus* subgenus and identifying selective trait loci in edible cherries are crucial for the improvement of cherry cultivars to meet producer and consumer demands. In this study, we performed a de novo assembly of a chromosome-scale genome for the sweet cherry (*Prunus avium* L*.*) cultivar ‘Burlat’, covering 297.55 Mb and consisting of eight chromosomes with 33,756 protein-coding genes. The resequencing and population structural analysis of 384 *Cerasus* representative accessions revealed that they could be divided into four groups (Group 1, Group 2, Group 3, and Group 4). We inferred that Group 1 was the oldest population and Groups 2, 3, and 4 were clades derived from it. In addition, we found selective sweeps for fruit flavor and improved stress resistance in different varieties of edible cherries (*P. avium*, *P. cerasus*, and *P. pseudocerasus*)*.* Transcriptome analysis revealed significant differential expression of genes associated with key pathways, such as sucrose starch and sucrose metabolism, fructose and mannose metabolism, and the pentose phosphate pathway, between the leaves and fruits of *P. avium*. This study enhances the understanding of the evolutionary processes of the *Cerasus* subgenus and provides resources for functional genomics research and the improvement of edible cherries.

**Supplementary Information:**

The online version contains supplementary material available at 10.1186/s43897-024-00120-4.

## Core

We successfully assembled a chromosome-scale genome of *Prunus avium* (‘Burlat’), and identified 33,756 protein-coding genes. Population structure analysis of 384 *Cerasus* accessions revealed four distinct groups, with Group 1 identified as the oldest. Selective sweeps for traits like fruit flavor and stress resistance were detected in *P. avium*, *P. cerasus*, and *P. pseudocerasus*. Transcriptome analysis revealed significant differential expression of genes involved in key metabolic pathways, including sucrose, fructose, and mannose metabolism, between the leaves and fruits of *P. avium*. Our findings provide new insights into the evolutionary processes of the *Cerasus* subgenus and offer valuable genomic resources for the improvement of edible cherry cultivars.

### Gene and accession numbers

All sequence data and genome files generated for this study were deposited in NGDC (https://ngdc.cncb.ac.cn/gsa/) under Bio Project ID PRJCA024707.

## Introduction

*Cerasus*, a subgenus of the genus *Prunus* in the family Rosaceae, generically contains approximately 150 species with different ploidies ranging from 2 × to 4 × (Li and Li 2007). It is primarily distributed in the temperate regions of Asia, Europe, and North America (Zhang et al. 2021). Based on the application value requirements, the subgenus *Cerasus* is classified into three main categories: ornamental, edible, and medicinal. The key force behind this diversification is the selective breeding of *Cerasus* plants to meet the demands for fresh and processed products.

Despite the importance of *Cerasus* plant diversification and the economic value of cultivar improvement, their evolutionary relationship has been controversial. The current consensus is that the *Cerasus *subgenus originated in East Asia (i.e., China, Japan, Korea, and Russia), especially in western and eastern China and along Japan and Korea (i.e., Himalaya-Japan) (Chen et al. 2020; Li, 2007). However, the origins of sweet cherry and sour cherry (*Prunus cerasus*) are believed to be traced back to a region south of the Caucasian mountains around the Caspian and Black Seas before they were disseminated across Europe and Russia by explorers (Mariette et al. 2010). Given that natural interbreeding among the *Cerasus* subgenus is universal, it is unsurprising that its evolutionary relationships remain controversial. Otherwise, edible cherry varieties mainly belong to three *Cerasus* species, *P*. *avium*, *P. cerasus*, and *P. pseudocerasus*, and are highly valued by consumers owing to their fruit taste and nutritional quality, and by producers owing to their economic importance (Welk 2016). Recent genomic studies focusing on cultivated cherry and related species within the *Cerasus *subgenus have pinpointed specific genomic regions (Donkpegan et al. 2023; Holušová et al. 2023; Wang et al. 2023a, b) associated with fruit taste, flavor, color (Calle et al. 2020; Calle et al. 2021; Sooriyapathirana et al. 2010), size (Rosyara et al. 2013; Zhang et al. 2010; Campoy et al. 2015), firmness (Cai et al. 2019; Crump et al. 2022), and susceptibility to fruit cracking (Barreneche et al. 2021; Crump et al. 2022). Their findings indicated that these selection pressures were evident during both the domestication process and subsequent improvement efforts. However, substantial phenotypic variation exists among different edible cherry varieties, and their genetic basis remains unknown.

For a comprehensive understanding of the genetic relationships and selection signatures within the *Cerasus* subgenus, single nucleotide polymorphism (SNP) markers were utilized and identified through population-level whole-genome resequencing (WGR). Currently, WGR has been extensively applied to various crops, such as rice (Wang et al. 2018), maize (Grzybowski et al. 2023), grapes (Dong et al. 2023), apples (Liao et al. 2021), and other important economic crops (Yao et al. 2022). Previous studies have shown that resequencing analysis can be applied in exploring the population structure and genetic relationships of *Cerasus* subgenus accessions. Recently, 18 species (Cao et al. 2022; Zhu et al. 2023; Zheng et al. 2022; Baek et al. 2018; Fang et al. 2022; Wang et al. 2022; Goeckeritz et al. 2023; Shirasawa et al. 2021; Shirasawa et al. 2019; Jiu et al. 2023, 2024a, b) within the *Cerasus* subgenus were successively subjected to genome assembly, encompassing four *P. avium* cv. ‘Big Star’ (Pinosio et al. 2020), ‘Satonishiki’ (Shirasawa et al. 2017), ‘Tieton’ (Wang et al. 2020a); v2.0 (Wang et al. 2020b), and ‘Regina’ (Le 2020). Despite ongoing improvements in the quality of the reference genome assembly for sweet cherries, high-quality data for elite sweet cherry cultivars remains lacking.

In the present study, we combined Illumina, Nanopore and chromosome conformation capture (Hi-C) data to assemble a high-quality chromosomal genome of *P. avium*. Additionally, a WGR of 384 edible and ornamental cherry accessions (289 *P. avium*, six *P. mahaleb*, three *P. cerasoides*, eight *P. serrulata*, three *P. tomentosa*, 31 *P. cerasus*, 35 *P. pseudocerasus*, eight *P.* × *gondouinii*, and one *P. fruticosa*) was performed. With approximately 33 million high-quality SNPs, our results revealed the population structure and evolutionary relationships among species of the *Cerasus* subgenus. Furthermore, selective sweeps associated with agriculturally important genes were identified in some cultivated edible cherry species. Our findings provide genetic evidence regarding the evolutionary relationships of the *Cerasus* subgenus and facilitate trait selection for edible cherry breeding.

## Results

### De novo assembly of *P. avium* cv. ‘Burlat’ genome

**Fig. 1:**
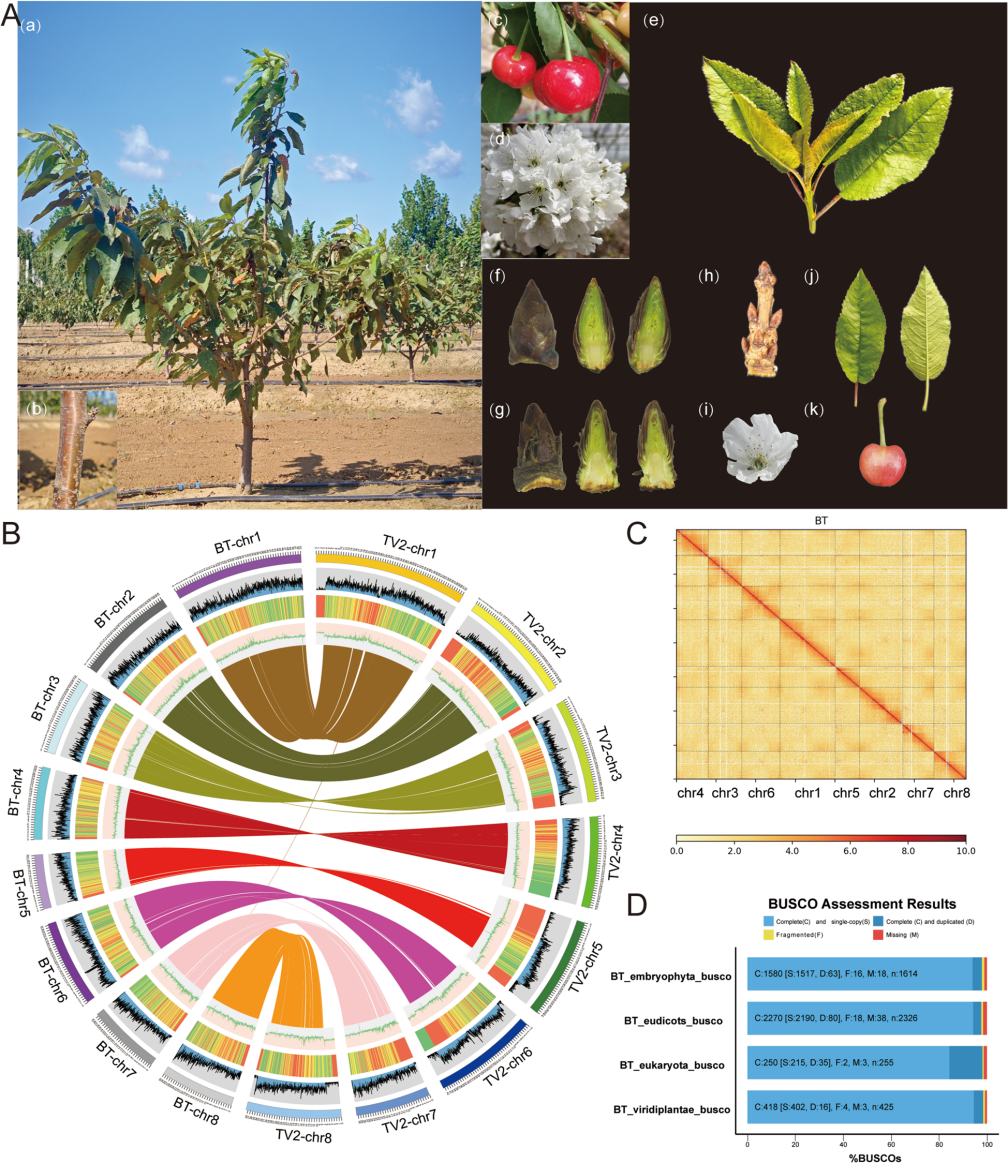
Characteristics of the *P. avium* cv. ‘Burlat’ genome. **A** Phenotypic characteristics of *P. avium* cv. ‘Burlat' trunks, flowers, leaf buds, flower buds, leaves, berries collected between February and May of 2022 and 2023. **B** Landscape of the *P. avium* genome assembly. The circles (outer to inner) represent chromosome ideogram (chr1 to chr 8), gene density, repetitive sequences density, GC content, and duplicated gene links between ‘Burlat’ and ‘Tieton v2’ genomes. Chromosomes are shown in a 50 kb window. **C** Hi-C contact map revealing extensive hierarchical chromatin interactions in the genome of *P. avium.* **D** Benchmarking Universal Single-Copy Orthologs (BUSCO) assessment of the completeness of the *P. avium* genome

The elite sweet cherry cultivar ‘Burlat’ was selected for genome sequencing and assembly (Fig. 1A). The genome size of *P. avium* was estimated based on a 17–31-mer distribution analysis, which was less than the previous k-mer-based estimate for *P. avium* cv. ‘Big Star’ (322 Mb) (Pinosio et al. 2020), ‘Satonishiki’ (352.9 Mb) (Shirasawa et al. 2017) and ‘Tieton’ (341.38 Mb) (Wang et al. 2020) (Figure S1, Tables S1, S2, and S5). By integrating 192 Gb of long reads from the Nanopore platform (Figure S2) and 84.71 Gb of short-read sequence data from the Illumina NovaSeq (Table S1), we yielded 298.03 Mb of the chromosome-level genome sequences with a scaffold N50 length of 32.05 Mb. A total of 273.34 Mb (~ 91.72%) were anchored to eight pseudochromosomes within the genome (Table S3, Figs. 1B, 1C, S3, and S4). To assess integrity and accuracy, 99.06% of Illumina short reads were mapped to the assembly, covering 99.0% of the genome. Bench-marking Universal Single-Copy Orthologs (BUSCO) analysis of 1,614 conserved single-copy plant genes revealed 97.9% completeness with only 18 genes missing in the assembly, which was comparable to ~ 97.9% for the cherry ‘Tieton v2’ (Wang et al. 2020) assembly (Fig.1D and S5, Table S4).

### Annotation of *P. avium* cv. ‘Burlat’ genome

We predicted 133.76 Mb of repetitive sequences, accounting for 48.94% of the assembled genome, including 32.63% retrotransposons and 9.55% DNA transposons. Long terminal repeat (LTR) retrotransposons were the major components, containing 84.32 Mb sequences, and accounted for 30.85% of the genome, with 17.25% *Gypsy* and 12.50% *Copia* (Table S6). Genome annotation revealed 33,756 protein-coding genes. The average length, number of introns, and number of exons per gene were 2,915 bp, 5.47, and 4.47, respectively (Table S7). BUSCO analysis showed that 98.4% of the gene set was completely covered by the genome annotations (Table S9 and Figure S5). In addition, 33,131 (98.15%) genes were functionally annotated using nine public databases: Non-Redundant Protein Sequence (NR) (33,125), eggNOG (28,222), Swiss-Prot (22,868), Pfam (23,071), Clusters of Orthologous Groups of Proteins (COG) (28,222), Gene Ontology (GO) (10,328), Kyoto Encyclopedia of Genes and Genomes (KEGG) (12,987), the *Arabidopsis* Information Resource (TAIR) (25,864), and the *Oryza sativa* Information Resource (MSU) (26,896) (Table S8).

### Evolutionary relationships and population structure in *Prunus* subgenus* Cerasus*

**Fig. 2:**
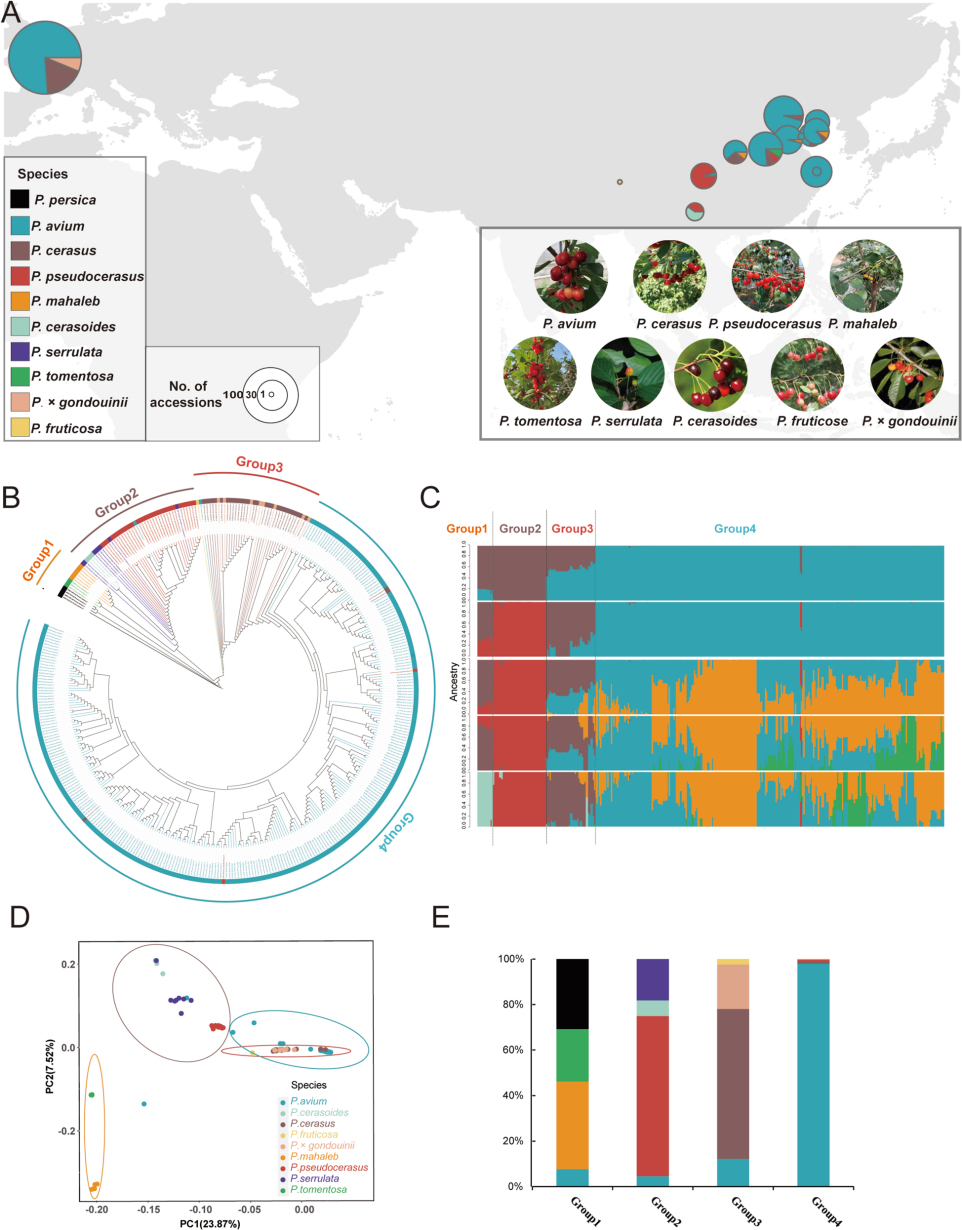
Population structure and geographic distribution of the 384 accessions of *Cerasus* subgenus germplasm. **A** Geographic locations and fruit phenotypes of representative varieties from 384 *Cerasus* accessions. The size of each pie chart represents the sample size. The fruit images of *P. cerasoides*, *P. fruticosa*, and *P.* × *gondouinii* were sourced from external references. *P. cerasoides* fruit image adapted from Gao Xinxin, sourced from https://ppbc.iplant.cn/tu/2594155; *P. fruticosa* fruit image adapted from Gao Xiaohui, sourced from https://ppbc.iplant.cn/tu/653724; *P.* × *gondouinii* fruit image adapted from the article "Analysis of agromorphological between duke cherry (*P.* × *gondouinii* (Poit. & Turpin) Rehd.) and its progenitors: sweet cherry (*Prunus avium* L.) and sour cherry (*Prunus cerasus* L.)". **B** Maximum likelihood phylogenetic tree for 384 accessions based on SNPs. Four *P. persica* accessions were used as the outgroup species. **C** Model-based clustering analysis with different cluster numbers (k = 2–6). The *y*-axis quantifies cluster membership, and the *x*-axis lists the different accessions. The orders and positions of these accessions on the *x*-axis are consistent with those presented in the tree. **D** Principal component analysis of the first two components of the 384 accessions. **E** Histogram of species distribution of the accessions in each group

The evolutionary relationships among the various species of the *Cerasus* subgenus remain controversial. To obtain the genotypes of these germplasm resources, 3.96 Tb of whole-genome sequencing data (13,205,172,192 paired-end raw reads) were generated for the 384 accessions sampled from nine *Cerasus* species (Fig. 2A and Table S10). After applying basic filtering criteria, the reads were mapped onto the assembled *P. avium* cv. ‘Burlat’ genome. The average alignment rate was 95.36% (82.31%–98.17%). The sequencing quality, alignment rate, and sequence depth of the reads among different individuals are shown in Table S10. After the identification of SNPs and indels using the Genome Analysis Toolkit (GATK), a basic set containing 33,790,533 SNPs and 4,538,846 indels was obtained (Table S11).

To decipher the genetic relationships among these species, we employed maximum likelihood (ML)-based phylogenetic analysis using whole-genome SNPs from the 384 accessions, with the closely related *P. persica* serving as an outgroup. The phylogenetic tree (Fig. 2B) classified the species into four primary clades, i.e., Group 1, *P. tomentosa* and *P. mahaleb*; Group 2, *P. serrulata*, *P. cerasoides*, and *P. pseudocerasus*; Group 3, *P. cerasus*,* P.* × *gondouinii*, and *P. fruticosa*; and Group 4, *P. avium*. The findings were consistent with the population structure results (Fig. 2C and S6; K = 5). This topology corroborated previous findings (Wan et al. 2023), indicating that *P. tomentosa* shares a sister relationship with other clades. Among the remaining species, *P. mahaleb* formed a sister clade with Group 2, which collectively constituted a sister clade with Groups 3 and 4. Interestingly, the Group 3 topology suggests the possibility of gene flow between the interrelated *P. cerasus* and *P.* × *gondouinii* species. A few accessions were grouped into clades with different genetic backgrounds (Fig. 2B). For instance, the sweet cherry accession SH1 (‘Krymsky No.5') was positioned within Group 3, whereas TA15 (‘Kale') exhibited clustering within Group 2. A possible explanation is potential misclassification based on morphology during sample collection. Another plausible interpretation is the presence of interspecific hybridization in the germplasm samples.

Principal components analysis (PCA) revealed the presence of four clusters, with the first three principal components explaining 23.87%, 7.52%, and 6.45% of the total genetic variance (Figs. 2D and S7). Among them, PC1 separated Groups 1 and 2 from Groups 3 and 4, suggesting that the latter two groups shared more similarities in genetic background than Groups 1 and 2. This finding was further supported by the results of the phylogenetic analysis (Fig. 2B). PC2 clearly segregated the samples in Group 1 into two species, *P. tomentosa* and *P. mahaleb*, and delineated the samples in Group 2 into three species, *P. pseudocerasus*, *P. cerasoides*, and *P. serrulata* (Fig. 2E), which originated in the southwestern region of China and subsequently expanded to other areas (Zhang et al. 2021; Chen et al. 2020).

### Demographic history and introgression in *Cerasus* subgenus

**Fig. 3:**
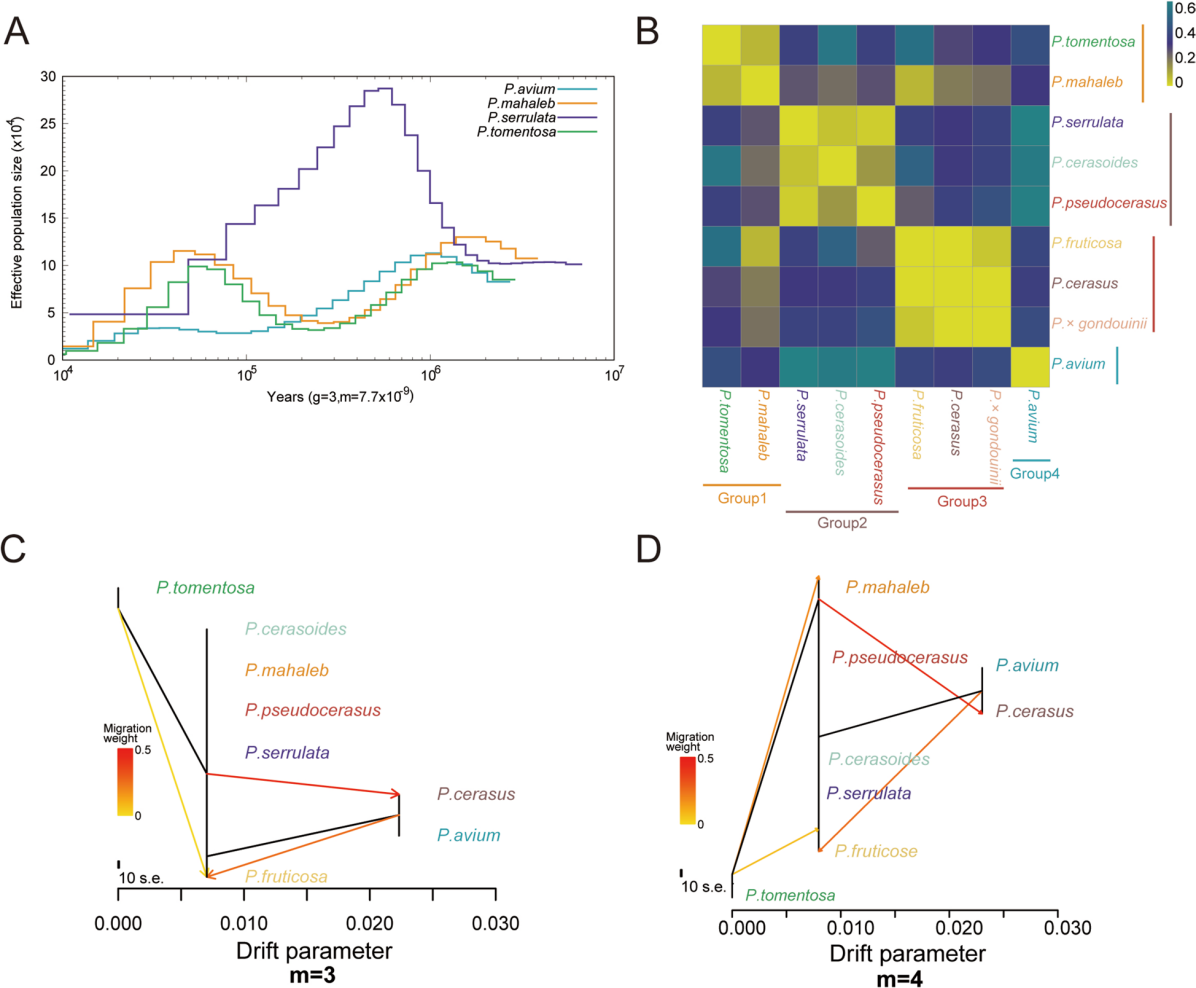
Demographic history of *Cerasus* subgenus species. **A** Demographic history of diploid *Cerasus* species (*P. avium*, *P. mahaleb*, *P. serrulata*, and *P. tomentosa*) germplasm inferred from the estimation of the historical effective population size (*Ne*) using the pairwise sequentially Markovian coalescent method. **B** Genetic differentiation (*F*_*ST*_) within nine *Cerasus* subgenus species. **C–D** Population splits and migrations among *Cerasus* species. Colored lines represent gene flows, and arrows indicate the direction of the gene flow

To explore the demographic history of *Prunus* subgenus *Cerasus*, we estimated the historical effective population size (*Ne*) of their ancestors using the PSMC method (Li and Durbin 2011) based on whole-genome SNPs. Previous studies have shown that the demographic history of perennial fruit crops, such as peaches (Yu et al. 2021), apples (Liao et al. 2021), and grapes (Liang et al. 2019), typically exhibits a pattern of sequential contraction and expansion in *Ne*. However, our study revealed a more complex scenario in the *Cerasus* subgenus populations comprising nine species (Figs. 3A and S8). Demographic history analysis indicated that five species, *P. tomentosa*, *P. mahaleb*, *P. avium*, *P. fruticosa*, and *P. cerasus*, experienced an initial bottleneck during the Pleistocene glaciation (1.17 to 0.8 million years ago). Subsequently, a second bottleneck occurred approximately 70,000 years ago during the last glaciation (70.0 to 11.5 thousand years ago) in two *Cerasus* subgenus species: *P. mahaleb* and *P. tomentosa* (Fig. 3A). This finding is consistent with the effective population bottleneck observed in perennial strawberries (Qiao et al. 2021)_._ Notably, *P. fruticosa*, *P. cerasus*, and *P. avium*, which originate from the Asia Minor Peninsula (Pervaiz et al. 2015), displayed relatively limited effects from the Last Glacial Maximum (LGM) and exhibited varying degrees of effective population expansion, particularly *P. fruticosa* and *P. cerasus*. This anomaly may be attributed to the comparatively minor influence on the Asia Minor Peninsula during the LGM (Yi 2018). Additionally, this pattern, particularly *Ne* contraction, was not observed during the Pleistocene in *P. cerasoides*, *P.* × *gondouinii*, and *P. pseudocerasus *(Figures S8a, b, and e). A probable explanation is that these species are predominantly distributed in tropical and subtropical regions near the equator. The influence of the LGM, which resulted in reduced temperatures and altered precipitation patterns, paradoxically promoted plant growth (Lü et al. 2018).

To study genetic divergence, we calculated the genetic distance (*F*_*ST*_) between species (Fig. 3B). The results demonstrated minimal genetic divergence within groups and substantial differentiation among groups (Fig. 3B), which is consistent with the results of both the phylogenetic analysis and population structure. A strong ancestral gene flow was identified from the roots (except for *P. tomentosa*) to *P. cerasus* and from the roots of *P. avium* and *P. cerasus* to *P. fruticosa* (Fig. 3C, D). Gene flow between these species may have been important in facilitating the rapid diversification of the *Cerasus* subgenus.

### Pedigree analysis within *Cerasus* subgenus accessions

**Fig. 4:**
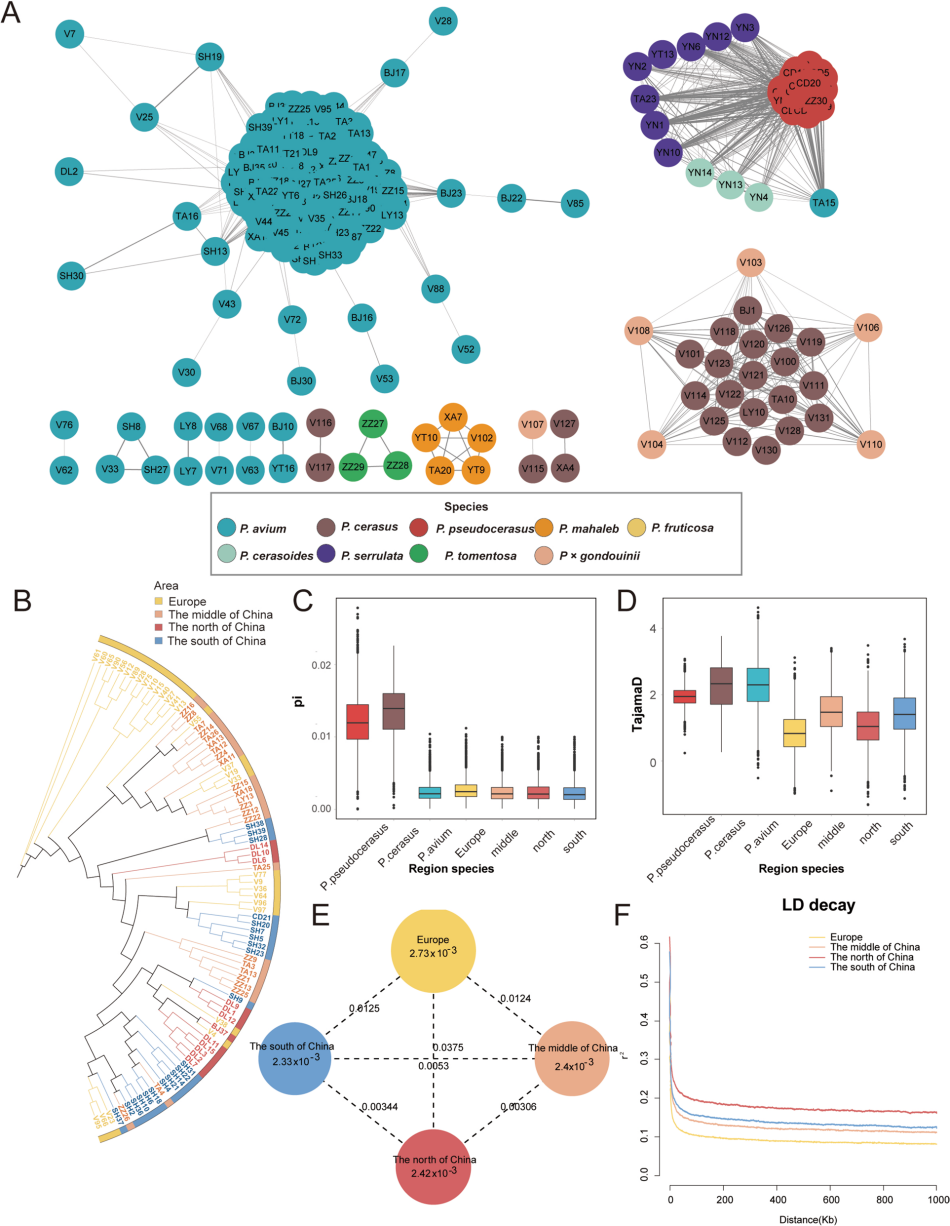
Genetic diversity and differentiation across *Cerasus* and *P. avium* accessions from different geographic regions. **A** Degree relationship network of 384 *Cerasus* accessions. The shades of line color are proportional to the calculated identity-by-descent (IBD) values. **B** Phylogenetic tree of 87 *P. avium* accessions from different geographic regions after IBD filtering. **C** Nucleotide diversity (*π*) estimation from different geographic regions. **D** Tajima’s D estimation from different geographic regions. **E** Nucleotide diversity (*π*) and genetic differentiation (*F*_*ST*_) within different geographic regions calculated using the sliding window approach (100 kb windows with 100 kb steps). The circle size represents the mean value of *π* in each region. The numbers marked between each regions indicate the mean value of *F*_*ST*_. **F** Linkage disequilibrium decay among different regions

To investigate the pedigree relationships of the 384 *Cerasus* subgenus accessions, we analyzed the patterns of identity-by-descent (IBD) relationships among the accessions. The pedigree network constructed using Cytoscape showed that the majority of relationships were among cherry accessions (Fig. 4A). *P. avium* formed a compact standalone cluster, whereas *P. pseudocerasus* was highly connected to *P. cerasoides* and *P. serrulata*, consistent with the distribution of the population structure (Fig. 2B: Group 2). A plausible explanation for this finding is that the three *Cerasus* subgenus species may share a common ancestor, with origins and distribution primarily located in regions, including the Himalayan Mountain range. Additionally, a cluster was formed between *P. cerasus* and *P.* × *gondouinii*, whereas *P. mahaleb* and *P. tomentosa* constituted two separate clusters. Notably, a limited number of species constituted a loosely connected cluster (V76-V62, SH8-V33, and V116-V117), indicating that there remains untapped potential within this species for the development of hybrid cultivars.

### Genetic diversity and genetic structure distribution of *P. avium*

After filtering based on IBD, we identified 14,048,694 genome-wide genetic variants in the 141 edible cherry accessions, including 11,875,331 SNPs and 2,173,363 indels (Figure S9, Tables S12 and S13). The number of SNP variants per individual was significantly different (Mann–Whitney, *P* < 0.001) between sweet cherries and other edible cherries (*P. cerasus* and *P. pseudocerasus*). Conversely, the number of SNP variants per individual showed no significant difference (Mann–Whitney, *P* < 0.001) within the China–Europe region (Table S14), which is consistent with the results presented in Tables S15, S16, and Figure S10.

To investigate the genetic structure and distribution of sweet cherries in China and Europe, we employed the ML method to construct a phylogenetic tree for the 87 distinct genotypes of sweet cherry cultivars. Phylogenetic analysis of the different varieties showed no significant geographic distribution differences within the *P. avium* cultivated population in China (Figs. 4B and S11). The limited duration of introduction and cultivation has prevented the development of a unique genetic structure distribution among the diverse regions in China, corroborating the historical account of the introduction of sweet cherry varieties to China in 1871 (Zhang 2017).

We analyzed genetic diversity parameters, including nucleotide diversity (*π*), Tajima's D, observed heterozygosity (*Ho*), expected heterozygosity (*He*), inbreeding coefficient (*F*), and linkage disequilibrium (LD). After correcting for subpopulation sample sizes, *P. avium* had lower *π* (2.44 × 10^–3^) than other edible cherries (*π*_*P. pseudocerasus*_ = 1.2 × 10^–2^ and *π*_*P. cerasus*_ = 1.3 × 10^–2^) (Fig. 4C and Table S17). This observation is consistent with the conclusions derived from *He*, *Ho*, and *F* which indicated that *P. avium* displays the highest coefficient of inbreeding while exhibiting the lowest levels of *He* and *Ho* (Figure S12 and Table S18). This low level of diversity in *P. avium* is also evident when compared with other fruit species in the Rosaceae family, such as apricot (*P. armeniaca*) (*π* = 6.18 × 10^–3^) (Zhang et al. 2021), pear (*Pyrus*) (*π* = 5.5 × 10^–3^) (Wu et al. 2018), and peach (*P. persica*) (*π* = 3.41 × 10^–3^) (Cao et al. 2014). This might be attributed to the specific breeding methods (grafting and cutting) and reproductive traits associated with the domestication of sweet cherries. Among sweet cherry varieties, the European varieties exhibited a higher *π* (2.7 × 10^–3^) than varieties from southern (*π* = 2.40 × 10^–3^), northern (*π* = 2.33 × 10^–3^), and central (*π* = 2.42 × 10^–3^) China. The similar genetic diversity (Fig. 4C) suggests a relatively consistent introduction time for sweet cherries in the southern, northern, and central regions of China. The Tajima's D values displayed the following trend: middle China (1.50) > southern China (1.42) > northern China (1.04) > Europe (0.85) (Fig. 4D), mirroring the *π* analysis results and signifying a greater extent of selection within China than in Europe. Furthermore, in European cultivated varieties, the genetic diversity of the French and Spanish varieties was higher than that of the Italian varieties (Figure S13).

We further investigated the genetic divergence among regional subpopulations of *P. avium* (Fig. 4E and Table S19). The European subpopulation was genetically more distant, with greater genetic diversity than the Chinese subpopulations (southern, northern, and middle China), implying distinct genetic differentiation between Eurasian regions. The combined diversity and phylogeny analyses suggested that the ancestral populations of French and Spanish cherries may be more closely related to wild cherries (Figures S11 and S13). Additionally, *F*_*ST*_ values between the Chinese subpopulations indicated weaker genetic differentiation among regional populations. This could be attributed to the relatively short period since the introduction of sweet cherry into China or the relatively frequent gene exchange during the modern cultivation process in China.

Because LD decay is often associated with genetic diversity, we further analyzed the average LD values based on r^2^ among different species. The results showed that LD _*P. avium*_ > LD _*P. pseudocerasus*_ > LD _*P. cerasus*_, which was consistent with the results of nucleotide diversity, π _*P. avium*_ < π_*P. pseudocerasus*_ < π_*P. cerasus*_, and is primarily influenced by artificial selection (Figure S14). The distribution of r^2^/2 values for different regions of *P. avium* (Fig. 4F) was in the following order: Europe (0.2741) (0.197 kb) < central China (0.3329) (0.198 kb) < northern China (0.3145) (0.256 kb) < southern China (0.3154) (0.295 kb).

### Selective sweeps for *Cerasus* species

To gain insights into the genetic selection driving the differentiation of cultivated *Cerasus* species, we aimed to identify key selective signals during the evolution of *Cerasus* species. By analyzing genomic regions with marked differences in *π* and *F*_*ST*_ values (both top 5%) across comparisons between Group 1 and other groups, we detected 22 selective sweep regions between Groups 1 and 2, encompassing 10 candidate genes (Figure S15). Similarly, comparisons between Groups 1 and 3 revealed 138 selective sweep regions with 9 candidate genes (Figure S16), while the comparison between Groups 1 and 4 identified 142 selective sweep regions containing 8 candidate genes (Figure S17 and Table S20). Based on intergroup selective sweep analysis, we characterized the distinct evolutionary traits of the groups. Group 1, consisting of *P. tomentosa* and *P. mahaleb*, retained ancestral traits such as simple flower structures (Potter et al. 2007), indicating early divergence in *Cerasus* evolution. Group 2, which includes *P. serrulata*, *P. pseudocerasus*, and *P. cerasoides*, has evolved more derived traits like vivid flower coloration, probably as adaptations to new pollinators or varied environments. Selective sweeps between Groups 1 and 2 revealed genes associated with flower morphology, such as *PAV05G026040*, which encodes dihydroflavonol 4-reductase, an enzyme that converts dihydroflavonol to dihydroflavonone and plays a crucial role in flower color formation (Luo et al. 2016). Group 3, composed of *P. fruticosa*, *P. cerasus*, and *P.* × *gondouinii*, was characterized by diverse fruit traits and enhanced disease resistance, which likely facilitated adaptation to harsher conditions. The selective sweep analysis in Group 3 identified three disease resistance genes, *PAV01G006060*, *PAV03G002600*, and *PAV04G004330*, further supporting this possibility. Finally, Group 4, represented by sweet cherry, has undergone significant artificial selection, leading to larger, sweeter fruits. The selective sweep analysis of Group 4 also identified a gene related to fruit sweetness (*PAV03G048310*) (Table S20), which encodes the bHLH transcription factor. The previous studies have indicated that this gene are involved in the regulation of soluble sugar and acid accumulation (Yu et al. 2021).

**Fig. 5:**
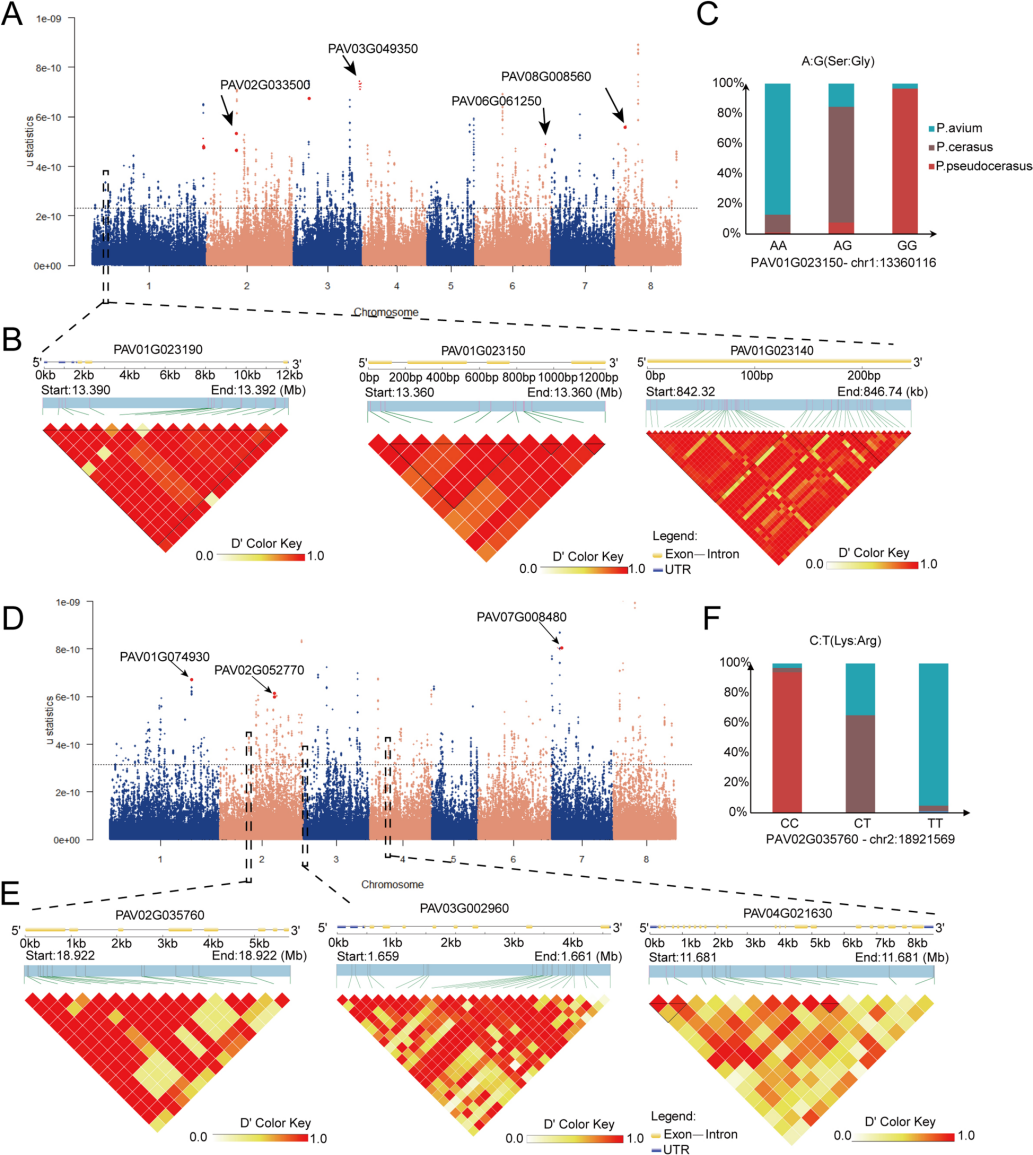
Identification of selection signatures and gene associations in edible cherries using RAiSD and linkage disequilibrium analysis. **A** μ statistics calculated by RAiSD across the genome in *P. cerasus*. The dashed lines mark the regions at the top 0.5%. The four non-synonymous mutated genes (*PAV02G033500*, *PAV03G049350*, *PAV06G061250*, and *PAV08G008560*) with the highest scores are labelled by arrows. **B** The gene structure (top) and linkage disequilibrium heat map (bottom) for genes associated with fructose metabolic process. The upper part: yellow rectangles and lines indicate exons and introns, respectively. **C** The bar plots for the candidate gene *PAV01G023150*, based on genotype. **D** μ statistics calculated by RAiSD across the genome in *P. pseudocerasus*. The three non-synonymous mutated genes (*PAV01G074930*, *PAV02G052770*, and *PAV07G008480*) with the highest scores are labelled by arrows. **E** The gene structure (top) and linkage disequilibrium heat map (bottom) for genes associated with regulation of innate immune response. **F** The bar plots for the candidate gene *PAV02G035760,* based on genotype

Following the identification of key selective sweeps, we further validated the candidate genes associated with selection signals in edible cherries (*P. avium*, *P. pseudocerasus*, and *P. cerasus*). By analyzing genomic regions (~ 1 kb in length) comprising the top 0.5% highest scores in μ statistical analysis (Figs. 5A, D, and S20; Tables S21, S23, and S25), we pinpointed selective signal genes across different cherry species. After filtering out synonymous and intronic mutations, we identified 183, 201, and 319 candidate genes in *P. pseudocerasus*, *P. avium*, and *P. cerasus*, respectively (Tables S22, S24, and S26). After filtering out synonymous and intronic mutant genes, 183, 201, and 319 candidate genes were selected from *P. pseudocerasus*, *P. avium*, and *P. cerasus*, respectively (Tables S22, S24, and S26).

GO analysis of *P. cerasus* revealed significant functional representation in immune response, regulation of cell death, fructose metabolic processes, and plasma membrane (Figure S18). Among these, the fructose metabolic process was the most significant, with three enriched genes identified. This implies a potential positive selection for fruit flavor associated with these genes. Among the three enriched genes, five non-synonymous SNPs were located in genes *PAV01G023140* and *PAV01G023190*, which encode the sugar transport protein *MST4 *(Wang et al. 2008; Wang et al. 2007), and *PAV01G023150*, which encodes the homolog of the sugar transport protein 13 (*STP13*) in *Arabidopsis *(Liu et al. 2016; Nuhse et al. 2004) (Fig.5B and Table S27). Notably, the function of the *MST4* gene as a monosaccharide transporter has been identified in rice, demonstrating its ability to transport glucose, fructose, mannose, and galactose (Wang et al. 2007). Therefore, this gene may influence the sweetness and acidity of sour cherry (*P. cerasus*). Genotypic analysis revealed a significant difference in haplotypes containing a non-synonymous SNP within the *PAV01G023150* gene among the different cherry species. In this haplotype, the SNP at position chr1:13,360,116 (C/T) resulted in an amino acid change from serine to glycine (Fig. 5C).

By comparison, GO enrichment analysis of *P. pseudocerasus* selection candidate genes showed significant functional representation associated with immune response, transmembrane transporter activity, and regulation of stress response (Figure S16), suggesting positive selection for resistance to biotic and abiotic stresses. Among the genes enriched in the regulation of the innate immune response, *PAV02G035760* encodes a RING/U-box superfamily protein. Previous studies identified RING/U-box proteins encoded by soybeans, and their expression levels are affected by drought stress (Zhang et al. 2017). Furthermore, *PAV03G002960* encodes the peroxisomal (S)-2-hydroxy-acid oxidase GLO4 (Esser et al. 2014), and *PAV04G021630* encodes the probable leucine-rich repeat receptor-like serine/threonine-protein kinase *At3g14840* (Marmagne et al. 2007; Gou et al. 2010). Among the three enriched genes, *PAV02G035760* contained a significant nonsynonymous SNP (T/C) located at 18,921,569 bp on chr2, leading to an amino acid change from lysine to arginine. Genotyping indicated that the *P. pseudocerasus* accessions with the homozygous mutation genotype were significantly less than those with the reference genotype (Fig. 5F).

Of the 201 selected candidate genes in *P. avium*, GO enrichment analysis revealed significant functional representation associated with spectrin, ion channel, and cytoskeletal protein binding and ATPase activity (Figure S21). Notably, the candidate gene, *PAV03G048310*, was identified in both the selective regions between groups 1 and 4, as well as in the selective region of the sweet cherry single population, indicating that this gene plays a critical role in the evolution of traits related to sugar and acid metabolism in sweet cherry. Another gene, *PAV01G084290*, encodes a transmembrane protein (*At3g47200*) which is involved in regulating skin thickness in soybeans (Qiao et al. 2022) (Table S27).

### Transcriptome analysis of glucose metabolism-related pathways and selected genes

**Fig. 6:**
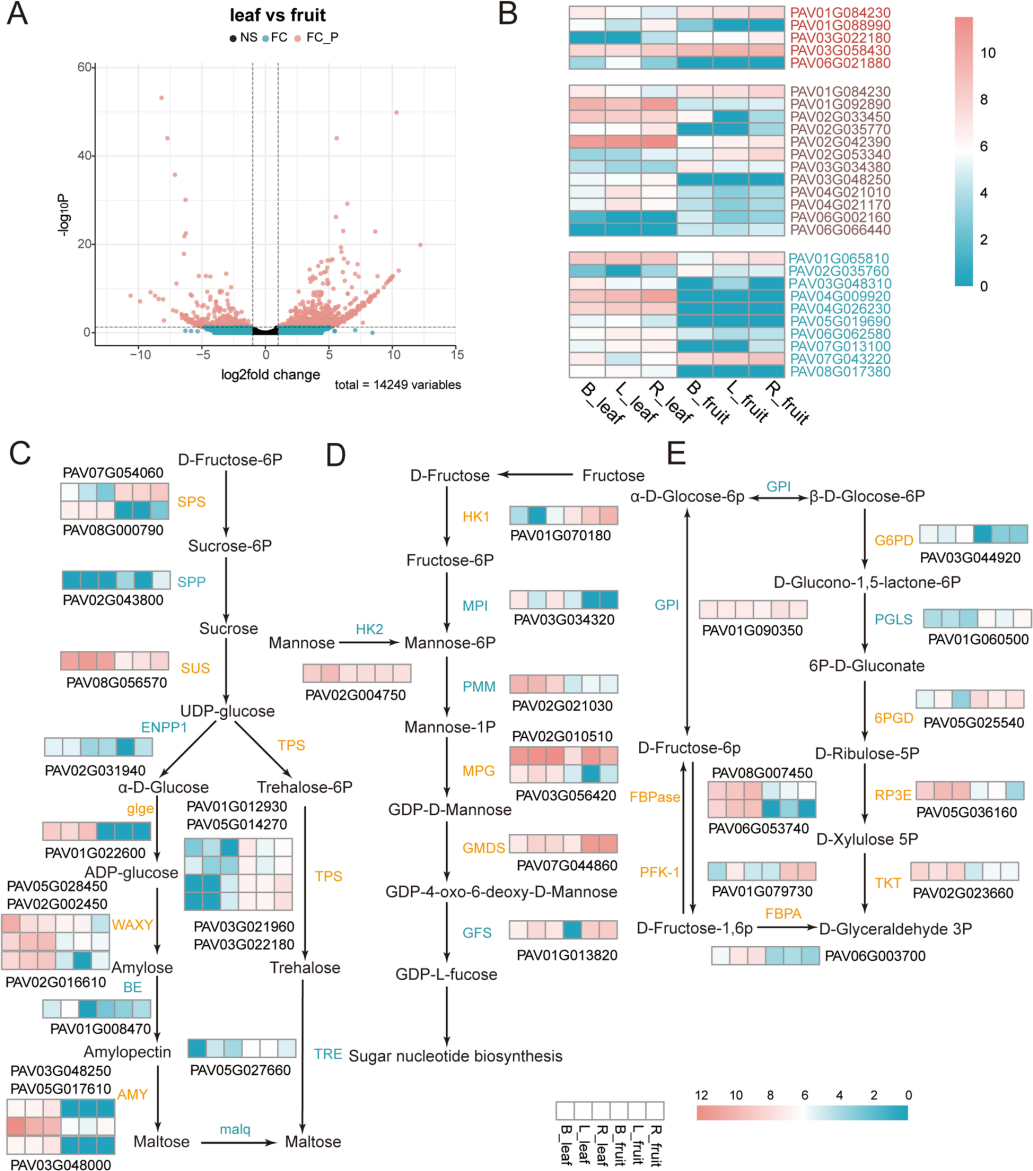
Differential gene expression and metabolic pathway changes between leaves and fruits in *P. avium*. **A** Differentially expressed genes. Two vertical lines indicate gene expression fold change (leaf vs fruit) > 2 and < 0.5 and the horizontal line indicates the adjusted P value of 0.05. Genes with significant differential expression (FC_P) are indicated by red dots (up-regulated and down-regulated); genes with no significant differential expression (NS) are represented by black dots; genes with differential expression and *p*-value > 0.05 (FC) are indicated by blue dots (up-regulated and down-regulated). **B** Heatmaps of edible cherry selective candidate differentially expressed genes. Among them, R_leaf, L_leaf, B_leaf and L_fruit, R_fruit, B_fruit refer to gene expression levels for leaves and fruits from three distinct *P. avium* cultivars (‘Bing’, ‘Lapins’ and ‘Rainier’). **C**–**E** DEGs involved in main sugar metabolic pathways in *P. avium* (leaf vs fruit). **C** Starch and sucrose metabolism. **D** Fructose and mannose metabolism. **E** Pentose phosphate pathway. Enzymes regulated by key DEGs are highlighted in yellow; Enzymes regulated by genes without significant differential expression are highlighted in blue

We identified 730 up-regulated and 373 down-regulated differentially expressed genes (DEGs) between fruits and leaves (Fig. 6A). By selecting candidate genes subjected to selective pressure within edible cherry populations, we analyzed the differential transcriptome expression between fruits and leaves, and identified five, 12, and 10 DEGs from candidate genes in *P. pseudocerasus*, *P. cerasus*, and *P. avium*, respectively (Fig. 6B).

To assess variations in the expression of sugar metabolism pathways in fruits and leaves, we identified 15 DEGs related to starch and sucrose metabolism through a comprehensive gene analysis (Fig. 6C). Of these, three *AMY1* (alpha-amylase), one *glge* (glucose-1-phosphate adenylyl transferase), one *SPS* (sucrose-phosphate synthase), one *SUS* (sucrose synthase), and three *WAXY* (granule-bound starch synthase) were upregulated in the leaf, whereas one *Ugp2* (UTP–glucose-1-phosphate uridylyl transferase), four *TPS* (alpha, alpha trehalose-phosphate synthase), and one *SPS* (sucrose-phosphate synthase) were upregulated in the fruit.

Meanwhile, we identified eight significant differences in genes related to fructose and mannose metabolism (Fig. 6D) and nine DEGs in the pentose phosphate pathway (Fig. 6E). In fructose and mannose metabolism, one *HK1* (Histidine kinase 1) and one *GMDS* (GDP-mannose 4,6 dehydratase) were upregulated in the fruit, whereas *MPG* was upregulated in the leaves. Additionally, in the pentose phosphate pathway, one *G6PD* (glucose-6-phosphate 1-dehydrogenase), one *FBPA* (fructose-bisphosphate aldolase), one *TKT* (transketolase), two *FBPase* (fructose-1,6-bisphosphatase), one *RP3E* (ribulose-phosphate-3-epimerase), and one *FBPA* (fructose-bisphosphate aldolase) were upregulated in the leaf, whereas one *6PGD* and *PFK-1* (6-phosphofructokinase 1) were upregulated in the fruits.

## Discussion

In this study, we used a *P. avium* cv. ‘Burlat’ genome assembly and resequencing of 384 diverse representative *Cerasus* subgenera accessions to reconstruct a detailed picture of the evolutionary history and genetic relationships of nine *Cerasus* species and identify the consequences of these selection processes for the genetic composition of economically important edible cherries. These resources will be valuable for further genomic research and facilitate iterative applications to explore the genetic diversity and evolutionary history of the *Cerasus* subgenus.

To study the genetic relationships among the nine *Cerasus* species and identify the genomic loci associated with the selective traits of edible cherries, we first performed a genetic relationship analysis based on a comprehensive dataset of whole-genome SNPs from 384 *Cerasus* accessions which divided the nine *Cerasus* species into four groups. Among the four groups, Group 1 (*P. tomentosa* and *P. mahaleb*) was the oldest population and closer to the root of the phylogenetic tree, whereas Groups 2 (*P. serrulata*, *P. cerasoides,* and *P. pseudocerasus*), 3 (*P. cerasus*, *P.* × *gondouinii*, and *P. fruticosa*), and 4 (*P. avium*) were clades derived from Group 1. This finding suggests possible genetic and evolutionary relationships among the nine *Cerasus* species. Populations of the subgenus *Cerasus* exhibited a complex demographic history characterized by four distinct evolutionary patterns. Although the limited sampling did not allow for the display of the whole scheme for the evolution of the nine *Cerasus* species, the results revealed important details regarding the demographic history of this subgenus. First, during the Pleistocene glaciation period (1.17 to 0.8 million years ago), the majority of *Cerasus* species experienced a contraction in *Ne* owing to the influence of climatic conditions. Second, during the LGM, approximately 80,000–20,000 years ago, the Earth underwent a cyclical alternation of multiple glacial and interglacial periods, resulting in pronounced climatic variations among different regions. This resulted in the tropical and subtropical regions experiencing glacier retreat due to factors, such as rising temperatures, climate warming, and human activities, leading to the rapid growth of *Cerasus* species (*P. cerasus*, *P.* × *gondouinii*, *P. fruticosa*, *P. pseudocerasus*, and *P. cerasoides*) in these regions. Conversely, the high-latitude and altitude areas in the Northern Hemisphere, characterized by perennial and extensive glacier coverage, exerted inhibitory effects on soil, vegetation, and plant survival. These conditions led to a secondary contraction in the *Ne* of *P. tomentosa* and *P. mahaleb*.

We identified that China-European genetic differentiations existed within sweet cherries, with European varieties exhibiting closer affinity to their wild relatives than those from China (Figs. 2B and 4B). The phylogenetic results showed a greater closeness of the central China group to the European root sweet cherry varieties (Fig. 4B), and there was less genetic divergence between the central China group and European sweet cherry varieties (Fig. 4E); suggesting that *P. avium* was probably introduced to the rest of China from central China (Shandong and Henan).

Phylogenetic analysis of the genetic structure distribution of sweet cherries indicated the absence of distinct genetic differentiation among the regions. Notably, as we moved from central China groups toward the northern and southern ones, the rate of LD decay gradually slowed. Similarly, nucleotide diversity gradually converged in central China towards both the northern and southern groups, and the changes in population differentiation indices slowly decreased. Collectively, these observations suggest a similar trend. One plausible explanation for these findings is that sweet cherries in China have experienced some level of regional differentiation owing to the influence of varying climatic conditions and environmental changes across different regions. Among these, the southern and northern groups exhibited a higher degree of selective breeding than the central group. However, the relatively short history of regional introduction and breeding programs has resulted in a less pronounced genetic differentiation between these regions. The genetic diversity in sweet cherry germplasm resources remains limited, and these constraints significantly impede further trait improvements and the development of new varieties. Therefore, future efforts should focus on the introduction of genetic resources from wild cherries and closely related species to ameliorate existing challenges in sweet cherry cultivation.

Analyses of the SNPs also shed light on a set of selective sweeps that promote edible cherry fruit flavor and resistance to biotic and abiotic stress. The results were consistent with the traits chosen during edible cherry selection. For instance, fruit acidity in the sour cherry (*P. cerasus*) is required to produce a nutrient-rich edible berry (Chunmei et al. 2011). Enrichment of genes controlling fruit flavor within the selective sweep regions identified in this study demonstrates that this trait plays an important role in the selection of sour cherry. Chinese cherry (*P. pseudocerasus*) has been selected for traits related to plant resistance to biotic and abiotic stresses (Ran et al. 2022; Hou 2022), which was consistent with the enrichment results of the selected candidate genes. While selective sweeps highlight key candidate genes involved in fruit flavor and stress resistance, the study is limited by the absence of experimental validation, particularly at the gene expression and functional levels. Addressing this gap in future work would provide more definitive insights into the biological roles of these candidate genes and further substantiate their contributions to cherry domestication.

In conclusion, our study elucidates the genetic relationships and demographic histories of nine *Cerasus* subgenus species and provides novel information on the distribution of genetic diversity of sweet cherries in Europe and China. Identification of interspecific genetic relationships within this subgenus offers a theoretical benchmark for investigating the genesis and development of *Cerasus* plants on a global scale. Furthermore, this extensive database of variations in *Cerasus* species facilitates future research on various aspects, such as pan-genomics, and functional genomics.

## Materials and methods

### Plant materials and DNA extraction

The sweet cherry cv. ‘Burlat’ originated from France and was selected for genome assembly. Young leaves of ‘Burlat’ were sampled from Tongchuan Haisheng Modern Agriculture Co., Ltd. in Shanxi, China (Latitude 108°50ʹ56ʺ N, 31°51ʹ14ʺ E). Fresh leaves of 384 *Cerasus* accessions from nine species (*P. mahaleb*, *P. cerasoides*, *P. serrulata*, *P. tomentosa*, *P. fruticosa*, *P.* × *gondouinii*, *P. avium*, *P. cerasus*, and *P. pseudocerasus*) were collected for resequencing analysis. During the sample selection, we acquired specimens from two key regions: eight *Cerasus* subgenus cultivation hubs in China and the well-known INRAE Prunus Genetic Resources Center in France (Table S10). The fresh leaves were placed on silica gel for rapid drying. Subsequently, DNA was extracted using the DNAsecure Plant Kit (Tiangen, Beijing, China), with 2 µg genomic DNA from each accession used for library construction. DNA purity, concentration, and integrity were assessed using a Qubit fluorescence spectrophotometer (Thermo Fisher Scientific, Waltham, MA, USA) and Agilent 2100 Bioanalyzer (Agilent Technologies, Santa Clara, CA, USA) according to the manufacturers’ instructions.

### Library construction and sequencing

Nanopore sequencing was performed using the Rapid Barcoding Kit/Rapid Sequencing Kit to construct libraries, which were sequenced on the PromethION Beta v19.05.1 platform (Oxford Nanopore Technologies, Oxford, UK), following the manufacturer’s recommendations. For genome-based correction and population genetic analysis, Illumina sequencing libraries were generated using an NEBNext Ultra DNA Kit (NEB Inc., Ipswich, MA, USA), according to the manufacturer’s instructions. Libraries were subjected to paired-end sequencing (PE150) using Illumina HiSeq X Ten (Illumina, San Diego, CA, USA) platform. Otherwise, a Hi-C library was also prepared using the following procedure: fresh, young leaves were first fixed in formaldehyde and lysed. The cross-linked DNA was then digested overnight with the four-cutter restriction enzyme Dpn II. The resulting fragments were ligated and biotinylated to form chimeric rings, which were subsequently enriched, sheared, and processed. The Hi-C library was sequenced on the Illumina HiSeq X Ten platform.

### Genome size estimation

To estimate genome size, Illumina reads were subjected to *k*-mer analysis using KmerGenie (Chikhi et al. 2014) (version 1.7051), where *k*-mer lengths ranged from 17 to 31 bp. The optimal *k*-mer length was chosen to predict genome size. Subsequently, *k*-mers were counted using Jellyfish (v.2.2.5) (Marçais et al. 2011), and the frequency spectrum was fitted using GnomeScope (v1.0.0) (Vurture et al. 2017). The genome size, heterozygosity, and repetitive sequence content of *P. avium* cv. ‘Burlat’ were estimated.

### Genome assembly and quality assessment

The long-read data generated by Oxford Nanopore were utilized for de novo genome assembly using NECAT-v0.0.1 (Chen 2020) (https://github.com/xiaochuanle/NECAT), a process encompassing raw read correction, contig assembly, and bridging contigs. The resultant assembled genome was refined by polishing using Racon3 (Vaser et al. 2017), with clean short reads utilized as the input to improve base accuracy. We conducted two rounds of purging using purge_dups-v1.2.5 (https://github.com/dfguan/purge_dups). Then, we performed Hi-C chromosome conformation analysis using Juicer v1.6.2 (Durand et al. 2016) and 3D-DNA v180922 (Dudchenko et al. 2017) with default settings. Subsequently, we evaluated the integrity of the Hi-C-assisted chromosomal assembly using Juicerbox v2.18.00 (https://github.com/aidenlab/Juicebox) and HicPlotter v0.6.6 (Akdemir and Chin 2015) to construct a chromatin contact matrix.

To assess the quality of the genome assembly, Illumina reads were aligned to the genome using the Burrows–Wheeler Aligner (BWA) (Li 2013) with default parameters to estimate the coverage ratio. The completeness and quality of the genome were evaluated using BUSCO v3.0.1 (Simão et al., 2015) with default parameters by mapping to the BUSCO library (embryophyta_odb10, eudicots_odb10, viridiplantae_odb10, and eukaryota_odb10).

### Genome annotation

Repeat sequences were annotated using de novo and homology-based approaches. For the de novo approach, RepeatModeler (version 1.0.4) (Flynn et al. 2020) was used to establish a repository of repeat elements. LTR sequences were identified using ltr_harvest (Ellinghaus et al. 2008), ltr_finder_parallel (Ou and Jiang 2019), and ltr_retriever (Ou et al. 2018), and a non-redundant LTR-RT library was generated. Finally, repetitive sequences were annotated using RepeatMasker (version 4.0.7) (Tarailo 2009). For homology-based annotation, the transposable elements were aligned against the RepBase library (RepBase 21.01) (Jurka et al. 2005) using RepeatMasker (Tarailo 2009) and RepeatProteinMask. Tandem Repeats Finder (version 4.0.9) (Benson and Benson 1999) was used to confirm tandem repeat sequences. Finally, the results from both de novo and homology-based annotations were integrated.

To annotate protein-coding gene structures in the genome, we utilized a combination of de novo prediction, protein evidence, and transcriptome evidence. For homology-based annotation, the protein sequences of *P. avium*(GCF_002207925.1) (Shirasawa et al. 2017) were aligned against the genome assembly using TBLASTN (v.2.2.26). Based on the aligned sequences, gene models were predicted using GeneWise v2.4.1 (Birney et al. 2004). For transcript-based annotation, RNA datasets from sweet cherry leaf and root tissues were obtained from the NCBI database (PRJNA595502, PRJNA419491, PRJNA550274, and PRJNA73727). All RNA reads from NCBI were aligned to the assembled genome using Tophat2 (Kim et al. 2013). The alignment results were used to construct transcripts using Cufflinks v2.1.1 (Trapnell et al. 2013), and the TransDecoder package (http://transdecoder.sourceforge.net/) was employed to predict open reading frames on the assembled transcripts. Subsequently, MIKADO 1.1 (Venturini et al. 2018) was utilized to filter and merge the genes obtained using two distinct approaches. For de novo gene prediction, we used the Augustus (Stanke et al. 2006) software to predict protein-coding genes. Ultimately, the outcomes of the three methods were amalgamated using MAKER2 (Holt and Yandell 2011).

BLAST (Altschul et al. 1990) with an e-value threshold of ≤ 1e-5 was used to annotate the relevant gene functions against nine local functional databases, including NR, UniProt Swiss-Prot, KEGG, COG, eggNOG, and two specialized protein databases for *Arabidopsis thaliana* and *Oryza sativa*. We utilized InterProScan and data from various sources, including Pfam and GO, to identify the GO and the gene model domains.

### Variation calling

All clean paired-end reads were aligned to the reference genome using BWA (v0.7.10-r789) (Li et al. 2009) with default parameters. Samtools v1.3.1 (Li et al. 2009) was then utilized to convert the mapping results into the BAM format and filter unmapped and non-unique reads. Duplicate reads were removed using Picard v2.1.1 (https://sourceforge.net/projects/picard/). After BWA alignment, realignment was conducted in two stages using GATK v3.3–0-g37228af (McKenna et al. 2010). Initially, the Realigner-Target-Creator package identified the regions requiring realignment. Subsequently, Indel Realigner was used to realign the regions identified in the preceding step, resulting in a realigned BAM file for each accession. Variant detection adhered to the best-practice workflow recommended by GATK. Variants were called for each accession using GATK HaplotypeCaller, and joint genotyping was performed on the gVCF files to merge variant alleles across different haplotypes.

During the filtering process, SNP filter criteria were established as QD < 5.0 || MQ < 40.0 || FS > 60.0 || SOR > 3.0 || MQRankSum < − 10.0 || ReadPosRankSum < − 8.0 || QUAL < 30, and the filter expression of Indel was set as QD < 2.0 || ReadPosRankSum < − 10.0 || InbreedingCoeff < − 0.8 || FS > 100.0 || SOR > 5.0 || QUAL < 30 (DePristo et al. 2011). Only insertions and deletions of 40 bp or shorter were considered. Subsequently, indels and SNPs with multiple alleles, > 50% missing rates, and MAF < 0.005 were removed, resulting in the creation of a basic set. SNPs with MAF < 0.05 were additionally excluded for phylogenetic tree structure analysis, IBD calculation, LD decay assessment, PCA, and population structure analyses, constituting the core set. Annotation of SNPs and indels was conducted using ANNOVAR v2015-12–14 (Wang et al. 2010), with the genome as a reference.

### Population genetics analysis

To investigate the evolutionary patterns among the sampled accessions, whole-genome SNPs were used to construct an ML phylogenetic tree with 100 bootstrap replicates using SNPhylo v20140701 (Lee et al. 2014). *P. persica* served as the outgroup for the analysis. The phylogenetic tree underwent color coding via the iTOL web server (https://itol.embl.de/).

Using PLINK v1.90b3.38 (Purcell et al. 2007), SNPs in LD were filtered out, with a window size of 50 SNPs (advancing 5 SNPs at a time) and an r^2^ threshold of 0.5. This process established a pruned SNP set for population structure analysis. LD-based pruning effectively mitigates the ascertainment bias (Malomane et al. 2018). GCTA v1.25.3 (Yang et al. 2011) was utilized to conduct PCA with subsequent plotting of the first three eigenvectors. PopLDdecay v3.41 (Zhang et al. 2019) was used to calculate LD, and ADMIXTURE v1.3 (Alexander et al. 2009) was utilized for analyzing population structure. We pre-specified the number of genetic clusters (K) from two to nine and performed a cross-validation error estimation to assess the convergence of individuals.

### Population evolutionary analysis

We employed TreeMix v1.13 (Pickrell 2012) to infer historical relationships among species, utilizing an ML approach based on a Gaussian model of allele frequency change. The topology of the ML trees varies depending on the number of migration events (m) allowed in the model, ranging from m = 3 to m = 4 (Fitak and Fitak 2021). The tree's bootstrap values were generated from 1,000 replicates.

To elucidate changes in the historical population size of the *Cerasus* subgenus species, we used PSMC (https://github.com/lh3/psmc) to infer the population size. Because of the propensity of PSMC to systematically underestimate actual event durations, particularly in scenarios of limited sequencing depth, we opted for samples exhibiting the highest mean coverage from each of the nine subgenera to guarantee the integrity of the consensus sequences. Using SAMtools v1.3.1 (Li et al. 2009), consensus sequences were generated and segmented into nonoverlapping 100-bp bins. The specified parameters included: -N25 -t15 -r5 -p '4 + 25 × 2 + 4 + 6'. A generation time of three years and a mutation rate of 7.7 × 10^–9^ per site per year (Xie et al. 1841) were used in the analysis.

### Pedigree construction

We computed the IBD for all pairwise comparisons within the 384 *Cerasus*accessions using PLINK v1.90b3.38 (Purcell et al. 2007). The lowest pairwise IBD value (≥ 0.5) were used as a stringent empirical cutoff. If the IBD value was > 95%, the pairs of accessions were considered genetically identical. A network figure based on IBD was generated using Cytoscape (version 3.10.0;http://www.cytoscape.org).

### Genetic diversity analysis

Vcftools v0.1.13 (Danecek et al. 2011) with 100 kb sliding windows was utilized to estimate genetic diversity, including*He*, *Ho*, *π*, and Tajima’s *D* using the filtered set of high-quality SNPs. Additionally, we employed the same software to compute the overall genetic differentiation across populations, quantified by Weir and Cockerham’s estimator of *F*_*ST*_.

### Detection of selected genes

RAiSD v2.9 (Raised Accuracy in Sweep Detection) (Alachiotis and Pavlidis 2018) was utilized to identify signatures of selective sweeps using the μ statistics, with a significance threshold set at the top 0.5% for the μ statistic score. Candidate-selected genes were identified based on genes annotated with non-synonymous SNP mutations found in these regions. GO enrichment analysis of the selected genes was conducted using the GO database. Then, we conducted a genome-wide investigation of selection signals by analyzing*F*_*ST*_ and *π*. Using a sliding window approach of 50 kb with 10 kb steps, we quantified *F*_*ST*_ and *π* with Vcftools v0.1.13 (Danecek et al. 2011). Genes annotated within regions exhibiting significant selection signals were identified as candidate loci under selection.

### Transcriptome analysis

To investigate the expression differences of selective genes and genes associated with sugar metabolic pathways in various tissues of edible cherries, we obtained transcriptomic data for leaves and fruits from three distinct *P. avium *cultivars (‘Bing’, ‘Lapins’, and ‘Rainier’) from the NCBI database (PRJNA497458) (Maldonado et al. 2019). DEGs were identified using the DESeq2 method (Love et al. 2014), and Reads Per Kilobase Million (RPKM) was used for gene-level quantification. Genes with adjusted |log_2_FC|> 2 and *p*-value < 0.05 were defined as DEGs. The R packages EnhancedVolcano and Pheatmap2 were employed to create an expression level heatmap and Volcano map for different tissues based on log_10_ RPKM data.

## Supplementary Information

Additional file 1: Figure S1. The 23-mer analysis of the *P. avium* genome. Figure S2. Frequency distribution of nanopore sequencing reads. Figure S3. High-resolution Hi-C interaction heatmap of *P. avium* genome. Figure S4. Summary of genome assembly and sequencing analysis of *P. avium*. (a) 8 chromosomes (chr. 1–8), (b) gene density, (c) GC content, and (d) Repeat sequence content in 50 kb sliding windows, and (e) synteny blocks among *P. avium* chromosomes. Figure S5. Comparison of BUSCO assessment of sweet cherry assemble and annotation quality; (a) Burlat sweet cherry genome; (b) Tieton-v2 sweet cherry genome; (c) Tieton-v1 sweet cherry genome. Figure S6. Cross-validation errors. The x-axis represents the K value, while y-axis indicates the cross-validation errors. The dot shows K = 6 with the lowest cross-validation errors. Figure S7. Principal component analysis of the first three components of the 384 accessions. Figure S8. Demographic history of Cerasus (a: *P. pseudocerasus*; b: *P* × *gondouinii*; c: *P. fruticose*, d: *P.cerasus* and e: *P. cerasoides*) germplasm inferred from the estimation of the historical effective population size *Ne* using the PSMC method. Figure S9. Diagram of SNPs found by resequencing of 141 individuals. Circles represent from outermost to innermost, (a) 8 chromosomes (chr. 1–8) denoted by different colors, (b) SNP abundance bars in improved populations, (c) INDEL abundance bars in improved populations and landraces, (d) nucleotide diversity abundance bars, (e) Tajama’D abundance bars. Figure S10. Venn diagrams for SNP variants detected (a) in *P. avium*, *P. cerasus* and *P. pseudocerasus*. (b) among different geographic regions. Figure S11. Phylogenetic tree of 87 *P. avium* accessions from different Eurasian geographic regions. Figure S12. Inbreeding coefficient, Proportion Observed Heterozygous Sites (%) and Proportion Expected Heterozygous Sites (%) estimation (a) in *P.avium*, *P.cerasus*, and *P. pseudocerasus* species; (b) among different geographic regions. Figure S13. Nucleotide diversity (*π*) and *tajama’D* estimation from different geographic regions. Figure S14. Linkage disequilibrium (LD) decay among edible cherries. Figure S15. Selective sweep regions during evolution of *Cerasus* inferred from *F*_*ST*_ and *ROD* statistics of group1 and group2. Figure S16. Selective sweep regions during evolution of *Cerasus* inferred from *F*_*ST*_ and *ROD* statistics of group1 and group3. Figure S17. Selective sweep regions during evolution of *Cerasus* inferred from *F*_*ST*_ and *ROD* statistics of group1 and group4. Figure S18. The enrichment analysis of GO in *P. cerasus*. Figure S19. The enrichment analysis of GO in *P.pseudocerasus*. Figure S20. μ statistics calculated by raisd across the genome in *P.avium*. The dashed lines mark the regions at the top 0.5%. Figure S21. The enrichment analysis of GO in *P.avium*.

Additional file 2: Table S1. Data statistics of sequence data used for *P.* *avium* genome assembly. Table S2. Genome survey of *P. avium* (kmer = 17–31). Table S3. Data statistics on 8 pseudomolecules for *P. avium*. Table S4. Completeness of the assembly measured by Benchmarking Universal Single-Copy Orthologs (BUSCO). Table S5. Comparisons of genome assembly for *P. avium*. Table S6. Classification and annotation of repetitive sequences. Table S7. Structural annotation of predicted genes. Table S8. Functional annotation of the predicted genes. Table S9. The annotated genes measured by Benchmarking Universal Single-Copy Orthologs (BUSCO). Table S10. Sequencing statistics for 384 *Cerasus* plant accessions. Table S11. Quantity table of indel and SNP after different filter conditions. Table S12. Sample statistics for edible cherries after IBD filtration. Table S13. Summary of snps and indels in edible cherry samples. Table S14. Statistical significance of differences in the number of variants among edible cherry species and different area by the Mann–Whitney test. Table S15. Distribution of SNP numbers between different varieties of edible cherries and different regions of sweet cherries. Table S16. Distribution of indel numbers between different varieties of edible cherries and different regions of sweet cherries. Table S17. *Tjama’D* and *π* among edible cherry species. Table S18. Observed heterozygosity, Expected heterozygosity, and *F* of edible cherries. Table S19. *F*_*ST*_ between regions of sweet cherries. Table S20. Functional annotation of inter-group selective candidate genes in *Cerasus* species. Table S21. Summary for *P. pseudocerasus* selective sweep regions from μ analysis. Table S22. Summary for exon variant in selective sweep regions for *P. pseudocerasus*. Table S23. Summary for *P. cerasus* selective sweep regions from μ analysis. Table S24. Summary for exon variant in selective sweep regions for *P. cerasus*. Table S25. Summary for *P. avium* selective sweep regions from μ analysis. Table S26. Summary for exon variant in selective sweep regions for *P. avium*. Table S27. Functional annotation of selection candidate genes for edible cherries.

## Data Availability

All sequence data and genome file generated for this study were deposited in NGDC (https://ngdc.cncb.ac.cn/gsa/) with Bio Project ID PRJCA024707.

## Abbreviations

BUSCO: Benchmarking Universal Single-Copy Ortholog
COG: Clusters of Orthologous Groups of Proteins
DEGs: Differentially expressed genes
GATK: The Genome Analysis Toolkit
GO: Gene Ontology
Hi-C: High-throughput chromosome conformation capture
KEGG: Kyoto Encyclopedia of Genes and Genomes
IBD: Identity-by-descent
LD decay: Linkage disequilibrium decay
LGM: Last Glacial Maximum
LTR: Long terminal repeat
ML: Maximum-likelihood
NR: Non-Redundant Protein Sequence
SNP: Single nucleotide polymorphism
PCA: Principal components analysis
WGR: Whole-genome resequencing
