# Correction: Population sequencing of cherry accessions unravels the evolution of *Cerasus* species and the selection of genetic characteristics in edible cherries

**DOI:** 10.1186/s43897-025-00148-0

**Published:** 2025-02-28

**Authors:** Yahui Lei, Songtao Jiu, Yan Xu, Baozheng Chen, Xiao Dong, Zhengxin Lv, Anthony Bernard, Xunju Liu, Lei Wang, Li Wang, Jiyuan Wang, Zhuo Zhang, Yuliang Cai, Wei Zheng, Xu Zhang, Fangdong Li, Hongwen Li, Congli Liu, Ming Li, Jing Wang, Jijun Zhu, Lei Peng, Teresa Barreneche, Fei Yu, Shiping Wang, Yang Dong, Dirlewanger Elisabeth, Shengchang Duan, Caixi Zhang

**Affiliations:** 1 Department of Plant Science, School of Agriculture and Biology, Shanghai Jiao Tong University, Shanghai, 200240 P. R. China; 2 College of Science, Yunnan Agricultural University, Kunming, Yunnan 650201 P. R. China; 3 College of Food Science and Technology, Yunnan Agricultural University, Kunming, 650201 Yunnan P. R. China; 4 UMR BFP, INRAE, Univ. Bordeaux, 71 Avenue Edouard Bourlaux, 33882 Villenave d’Ornon, France; 5 College of Horticulture, Northwest A&F University, Yangling, Shaanxi 712100 P. R. China; 6 Dalian Academy of Agricultural Sciences, Dalian, Liaoning 116036 P. R. China; 7 Yantai Academy of Agricultural Sciences, Yantai, Shandong 265500 P. R. China; 8 Horticulture Research Institute, Sichuan Academy of Agricultural Sciences, Sichuan, 610066 P. R. China; 9 Zhengzhou Fruit Tree Research Institute, Chinese Academy of Agricultural Sciences, Zhengzhou, Henan 450009 P. R. China; 10 Forestry and Fruit Research Institute, Beijing Academy of Agriculture and Forestry Sciences, Beijing, P. R. China; 11 Shanghai Botanical Garden, Shanghai, 200231 P. R. China; 12 College of Landscape and Horticulture, Yunnan Agricultural University, Kunming, Yunnan 650201 P. R. China; 13 Horticultural Research Institute, Yunnan Academy of Agricultural Sciences, Kunming, Yunnan 650201 P. R. China; 14 College of Plant Protection, Yunnan Agricultural University, Kunming, Yunnan 650201 P. R. China

**Correction** **:** **Mol Horticulture 5, 6 (2025)**

**https://doi.org/10.1186/s43897-024-00120-4**

Following publication of the original article (Lei et al. 2025), the authors reported that a few corrections were not implemented correctly by production, and thus need to be revised. The corrections are detailed as per below:

In the second paragraph of Introduction, a sentence was corrected from: 

“The current consensus is that the *Cerasus* subgenus originated in East Asia (i.e.,China, Japan, Korea, and Russia), especially in western and eastern China and along Japan and Korea (i.e., Himalaya-Japan) (Chen et al. 2020; (Li, 2007).”

To:

“The current consensus is that the *Cerasus* subgenus originated in East Asia (i.e., China, Japan, Korea, and Russia), especially in western and eastern China and along Japan and Korea (i.e., Himalaya-Japan) (Chen et al. 2020; Li, 2007).”

In the third paragraph of Introduction, two sentences were corrected from:

“Recently, (Jiu et al., 2024a, b) 18 species (Cao et al. 2022; Zhu et al. 2023; Zheng et al. 2022; Baek et al. 2018; Fang et al. 2022; Wang et al. 2022; Goeckeritz et al. 2023; Shirasawa et al. 2021; Shirasawa et al. 2019; Jiu et al. 2023) within the Cerasus subgenus were successively subjected to genome assembly, encompassing four P. avium cv. ‘Big Star’ (Pinosio et al. 2020), ‘Satonishiki’ (Shirasawa et al. 2017), ‘Tieton’ (Wang et al. 2020a); v2.0 (Wang et al. 2020b), and ‘Regina’ (Le 2020).”

“Despite ongoing improvements in the quality of the reference genome assembly for sweet cherries, high-quality data for elite sweet cherry cultivars.”

To:

“Recently, 18 species (Cao et al. 2022; Zhu et al. 2023; Zheng et al. 2022; Baek et al. 2018; Fang et al. 2022; Wang et al. 2022; Goeckeritz et al. 2023; Shirasawa et al. 2021; Shirasawa et al. 2019; Jiu et al. 2023, 2024a,b) within the Cerasus subgenus were successively subjected to genome assembly, encompassing four P. avium cv. ‘Big Star’ (Pinosio et al. 2020), ‘Satonishiki’ (Shirasawa et al. 2017), ‘Tieton’ (Wang et al. 2020a); v2.0 (Wang et al. 2020b), and ‘Regina’ (Le 2020).”

“Despite ongoing improvements in the quality of the reference genome assembly for sweet cherries, high-quality data for elite sweet cherry cultivars remains lacking.”

The legend of Fig. 3 was corrected from:

“**Fig. 3 **Demographic history of *Cerasus* subgenus species. **A** Demographic history of diploid *Cerasus* species (*P. avium*, *P. mahaleb*, *P. serrulata*, and *P.tomentosa*) germplasm inferred from the estimation of the historical effective population size (**Ne**) using the pairwise sequentially Markovian coalescent method. **B** Genetic differentiation (F_ST_) within nine *Cerasus* subgenus species. **C–D** Population splits and migrations among *Cerasus* species. Colored lines represent gene flows, and arrows indicate the direction of the gene flow”

To:

“**Fig. 3 **Demographic history of *Cerasus* subgenus species. **A** Demographic history of diploid *Cerasus* species (*P. avium*, *P. mahaleb*, *P. serrulata*, and *P.tomentosa*) germplasm inferred from the estimation of the historical effective population size (*Ne*) using the pairwise sequentially Markovian coalescent method. **B** Genetic differentiation (F_ST_) within nine *Cerasus* subgenus species. **C–D** Population splits and migrations among *Cerasus* species. Colored lines represent gene flows, and arrows indicate the direction of the gene flow”

The legend of Fig. 6 was corrected from: 

“**Fig. 6** Differential gene expression and metabolic pathway changes between leaves and fruits in *P. avium*. **A** Differentially expressed genes. Two vertical lines indicate gene expression fold change (leaf vs fruit)>2 and<0.5 and the horizontal line indicates the adjusted P value of 0.05. Genes with significant differential expression (FC_P) are indicated by red dots (up-regulated and down-regulated); genes with no significant differential expression (NS) are represented by black dots; genes with differential expression and **p-value>0.05 **(FC) are indicated by blue dots (up-regulated and down-regulated). **B **Heatmaps of edible cherry selective candidate differentially expressed genes. Among them, R_leaf, L_leaf, B_leaf and L_fruit, R_fruit, B_fruit refer to gene expression levels for leaves and fruits from three distinct *P. avium *cultivars (‘Bing’, ‘Lapins’ and‘Rainier’). **C–E** DEGs involved in main sugar metabolic pathways in P. avium (leaf vs fruit). **C** Starch and sucrose metabolism. **D** Fructose and mannose metabolism. **E** Pentose phosphate pathway. Enzymes regulated by key DEGs are highlighted in yellow; Enzymes regulated by genes without significant differential expression are highlighted in blue”

To:

“**Fig. 6** Differential gene expression and metabolic pathway changes between leaves and fruits in *P. avium*. **A** Differentially expressed genes. Two vertical lines indicate gene expression fold change (leaf vs fruit)>2 and<0.5 and the horizontal line indicates the adjusted P value of 0.05. Genes with significant differential expression (FC_P) are indicated by red dots (up-regulated and down-regulated); genes with no significant differential expression (NS) are represented by black dots; genes with differential expression and *p*-value>0.05 (FC) are indicated by blue dots (up-regulated and down-regulated). **B **Heatmaps of edible cherry selective candidate differentially expressed genes. Among them, R_leaf, L_leaf, B_leaf and L_fruit, R_fruit, B_fruit refer to gene expression levels for leaves and fruits from three distinct *P. avium *cultivars (‘Bing’, ‘Lapins’ and‘Rainier’). **C–E** DEGs involved in main sugar metabolic pathways in P. avium (leaf vs fruit). **C** Starch and sucrose metabolism. **D** Fructose and mannose metabolism. **E** Pentose phosphate pathway. Enzymes regulated by key DEGs are highlighted in yellow; Enzymes regulated by genes without significant differential expression are highlighted in blue”

In Additional file 2 of Supplementary Information, two sentences were corrected from: 

“Table S2. Genome survey of **P. avium** (kmer = 17–31).”

“Table S25. Summary for **P. avium** selective sweep regions from μ analysis.”

To: 

“Table S2. Genome survey of *P. avium* (kmer = 17–31).”

“Table S25. Summary for *P. avium* selective sweep regions from μ analysis.”

The below references have been corrected from:

“Liang Z, Duan S, Sheng J, Zhu S, Ni X, Shao J, et al. Whole-genome resequencing of 472 **Vitis** accessions for grapevine diversity and demographic history analyses. Nat Commun. 2019;10(1):1–12.”

“Qiao Q, Edger PP, Xue L, Qiong L, Lu J, Zhang Y, et al. Evolutionary history and pan-genome dynamics of strawberry (**Fragaria** spp.). Proc Natl Acad Sci. 2021;118(45):e2105431118.”

“Wang J, Liu W, Zhu D, Zhou X, Hong P, Zhao H, et al. A de novo assembly of the sweet cherry (*Prunus avium *cv. Tieton) genome using linked-read sequencing technology. PeerJ. 2020;8:e9114.”

“Wang J, Liu W, Zhu D, Hong P, Zhang S, Xiao S, et al. Chromosome-scale genome assembly of sweet cherry (*Prunus avium *L.) cv Tieton obtained using long-read and Hi-C sequencing. Hortic Res. 2020;7:122.”

“Wang Y, Xu Y, Xu J, Sun W, Lv Z, Manzoor MA, et al. Oxygenation alleviates waterlogging-caused damages to cherry rootstocks. Molecular Horticulture. 2023;3(1):1-23.”

“Wang J, Zhang X, Yan G, Zhou Y, Duan X, Wu C, et al. Development of a molecular marker for cherry crinkle leaf disease. Molecular Horticulture.2023;3(1):20.”

To:

“Liang Z, Duan S, Sheng J, Zhu S, Ni X, Shao J, et al. Whole-genome resequencing of 472 *Vitis* accessions for grapevine diversity and demographic history analyses. Nat Commun. 2019;10(1):1–12.”

“Qiao Q, Edger PP, Xue L, Qiong L, Lu J, Zhang Y, et al. Evolutionary history and pan-genome dynamics of strawberry (*Fragaria* spp.). Proc Natl Acad Sci. 2021;118(45):e2105431118.”

“Wang J, Liu W, Zhu D, Zhou X, Hong P, Zhao H, et al. A de novo assembly of the sweet cherry (*Prunus avium *cv. Tieton) genome using linked-read sequencing technology. PeerJ. 2020a;8:e9114.”

“Wang J, Liu W, Zhu D, Hong P, Zhang S, Xiao S, et al. Chromosome-scale genome assembly of sweet cherry (*Prunus avium *L.) cv Tieton obtained using long-read and Hi-C sequencing. Hortic Res. 2020b;7:122.”

“Wang Y, Xu Y, Xu J, Sun W, Lv Z, Manzoor MA, et al. Oxygenation alleviates waterlogging-caused damages to cherry rootstocks. Molecular Horticulture. 2023a;3(1):1-23.”

“Wang J, Zhang X, Yan G, Zhou Y, Duan X, Wu C, et al. Development of a molecular marker for cherry crinkle leaf disease. Molecular Horticulture.2023b;3(1):20.”

The original article (Lei et al. 2025) has been updated.
