# Supplementary material for: Population sequencing of cherry accessions unravels the evolution of Cerasus species and the selection of genetic characteristics in edible cherries
Source: Mol Hortic. 2025 Jan 8;5:6. doi: 10.1186/s43897-024-00120-4 (PMC11708008; doi:10.1186/s43897-024-00120-4)
Supplement: Supplementary file 1 — Additional file 1: Figure S1. The 23-mer analysis of the P. avium genome. Figure S2. Frequency distribution of nanopore sequencing reads. Figure S3. High-resolution Hi-C interaction heatmap of P. avium genome. Figure S4. Summary of genome assembly and sequencing analysis of P. avium. (a) 8 chromosomes (chr. 1–8), (b) gene density, (c) GC content, and (d) Repeat sequence content in 50 kb sliding windows, and (e) synteny blocks among P. avium chromosomes. Figure S5. Comparison of BUSCO assessment of sweet cherry assemble and annotation quality; (a) Burlat sweet cherry genome; (b) Tieton-v2 sweet cherry genome; (c) Tieton-v1 sweet cherry genome. Figure S6. Cross-validation errors. The x-axis represents the K value, while y-axis indicates the cross-validation errors. The dot shows K = 6 with the lowest cross-validation errors. Figure S7. Principal component analysis of the first three components of the 384 accessions. Figure S8. Demographic history of Cerasus (a: P. pseudocerasus; b: P × gondouinii; c: P. fruticose, d: P.cerasus and e: P. cerasoides) germplasm inferred from the estimation of the historical effective population size Ne using the PSMC method. Figure S9. Diagram of SNPs found by resequencing of 141 individuals. Circles represent from outermost to innermost, (a) 8 chromosomes (chr. 1–8) denoted by different colors, (b) SNP abundance bars in improved populations, (c) INDEL abundance bars in improved populations and landraces, (d) nucleotide diversity abundance bars, (e) Tajama’D abundance bars. Figure S10. Venn diagrams for SNP variants detected (a) in P. avium, P. cerasus and P. pseudocerasus. (b) among different geographic regions. Figure S11. Phylogenetic tree of 87 P. avium accessions from different Eurasian geographic regions. Figure S12. Inbreeding coefficient, Proportion Observed Heterozygous Sites (%) and Proportion Expected Heterozygous Sites (%) estimation (a) in P.avium, P.cerasus, and P. pseudocerasus species; (b) among different geographic regio [file 43897_2024_120_MOESM1_ESM.docx]

Supplemental information

**Population sequencing of cherry** **accessions unravels the evolution of** ***Cerasus* species and selection genetic** **characteristics in edible cherries**

Supplementary Figures and Tables

Supplemental information

**Supplementary Figure**

**Figure S1.** The 23-mer analysis of the *P. avium* genome

**Figure S2.** Frequency distribution of nanopore sequencing reads.

**Figure S3.** High-resolution Hi-C interaction heatmap of *P. avium* genome.

**Figure S4.** Summary of genome assembly and sequencing analysis of *P. avium*. **(a)** 8 chromosomes (chr. 1–8), **(b)** gene density, **(c)** GC content, and **(d)** Repeat sequence content in 50 kb sliding windows, and **(e)** synteny blocks among *P. avium* chromosomes.

**Figure S5.** Comparison of BUSCO assessment of sweet cherry assemble and annotation quality; **(a)** Burlat cherry; **(b)** Tieton-v2 cherry; **(c)** Tieton-v1 cherry.

**Figure S6.** Cross-validation errors. The x-axis represents the K value, while y-axis indicates the cross-validation errors. The dot shows K = 6 with the lowest cross-validation errors.

**Figure S7.** Principal component analysis of the first three components of the 384 accessions.

**Figure S8.** Demographic history of Cerasus (a: *P. pseudocerasus*; b: *P*× *gondouinii*; c: *P. fruticose*, d: *P.cerasus* and e: *P. cerasoides*) germplasm inferred from the estimation of the historical effective population size *Ne* using the PSMC method.

**Figure S9.** Diagram of SNPs found by resequencing of 141 individuals. Circles represent from outermost to innermost, (a) 8 chromosomes (chr. 1–8) denoted by different colors, (b) SNP abundance bars in improved populations, (c) INDEL abundance bars in improved populations and landraces, (d) nucleotide diversity abundance bars, (e) Tajama’D abundance bars.

**Figure S10.** Venn diagrams for SNP variants detected (a) in *P. avium*, *P. cerasus* and *P. pseudocerasus*. (b) among different geographic regions.

**Figure S11.** Phylogenetic tree of 87 *P. avium* accessions from different Eurasian geographic regions.

**Figure S12.** Inbreeding coefficient, Proportion Observed Heterozygous Sites (%) and Proportion Expected Heterozygous Sites (%) estimation (a) in *P. avium*, *P. cerasus*, and *P. pseudocerasus* species; (b) among different geographic regions.

**Figure S13.** Nucleotide diversity (*π*) and *tajama’D* estimation from different geographic regions.

**Figure S14.** Linkage disequilibrium (LD) decay among edible cherries.

**Figure S15.** Selective sweep regions during evolution of *Cerasus* inferred from *F_ST_* and *ROD* statistics of group1 and group2.

**Figure S16.** Selective sweep regions during evolution of *Cerasus* inferred from *F_ST_* and *ROD* statistics of group1 and group3.

**Figure S17.** Selective sweep regions during evolution of *Cerasus* inferred from *F_ST_* and *ROD* statistics of group1 and group4.

**Figure S18.** The enrichment analysis of GO in *P. cerasus*.

**Figure S19.** The enrichment analysis of GO in *P. pseudocerasus*.

**Figure S20.** μ statistics calculated by raisd across the genome in *P. avium*. The dashed lines mark the regions at the top 0.5%.

**Figure S21.** The enrichment analysis of GO in *P. avium*.

Supplementary Table

**Table S1.** Data statistics of sequence data used for *P. avium* genome assembly.

**Table S2.** Genome survey of Prunus avium (kmer=17-31)

**Table S3.** Data statistics on 8 pseudomolecules for *P. avium*.

**Table S4.** Completeness of the assembly measured by Benchmarking Universal Single-Copy Orthologs (BUSCO)

**Table S5.** Comparisons of genome assembly for *P. avium*.

**Table S6.** Classification and annotation of repetitive sequences.

**Table S7.** Structural annotation of predicted genes.

**Table S8.** Functional annotation of the predicted genes.

**Table S9.** The annotated genes measured by Benchmarking Universal Single-Copy Orthologs (BUSCO).

**Table S10.** Sequencing statistics for 384 *Cerasus* plant accessions.

**Table S11.** Quantity table of indel and SNP after different filter conditions.

**Table S12.** Sample statistics for edible cherries after IBD filtration.

**Table S13.** Summary of snps and indels in edible cherry samples.

**Table S14.** Statistical significance of differences in the number of variants among edible cherry species and different area by the Mann-Whitney test.

**Table S15.** Distribution of SNP numbers between different varieties of edible cherries and different regions of sweet cherries.

**Table S16.** Distribution of indel numbers between different varieties of edible cherries and different regions of sweet cherries.

**Table S17.** *Tjama’D* and *π* among edible cherry species.

**Table S18.** Observed heterozygosity, Expected heterozygosity, and *F* of edible cherries**.**

**Table S19.** *F_ST_* between regions of sweet cherries.

**Table S20.** Functional annotation of inter-group selective candidate genes in *Cerasus* species

**Table S21.** Summary for *P. pseudocerasus* selective sweep regions from μ analysis.

**Table S22.** Summary for exon variant in selective sweep regions for *P. pseudocerasus.*

**Table S23.** Summary for *P. cerasus* selective sweep regions from μ analysis.

**Table S24.** Summary for exon variant in selective sweep regions for *P. cerasus*.

**Table S25.** Summary for P. avium selective sweep regions from μ analysis.

**Table S26.** Summary for exon variant in selective sweep regions for *P. avium*.

**Table S27.** Functional annotation of selection candidate genes for edible cherries

Supplementary Figures


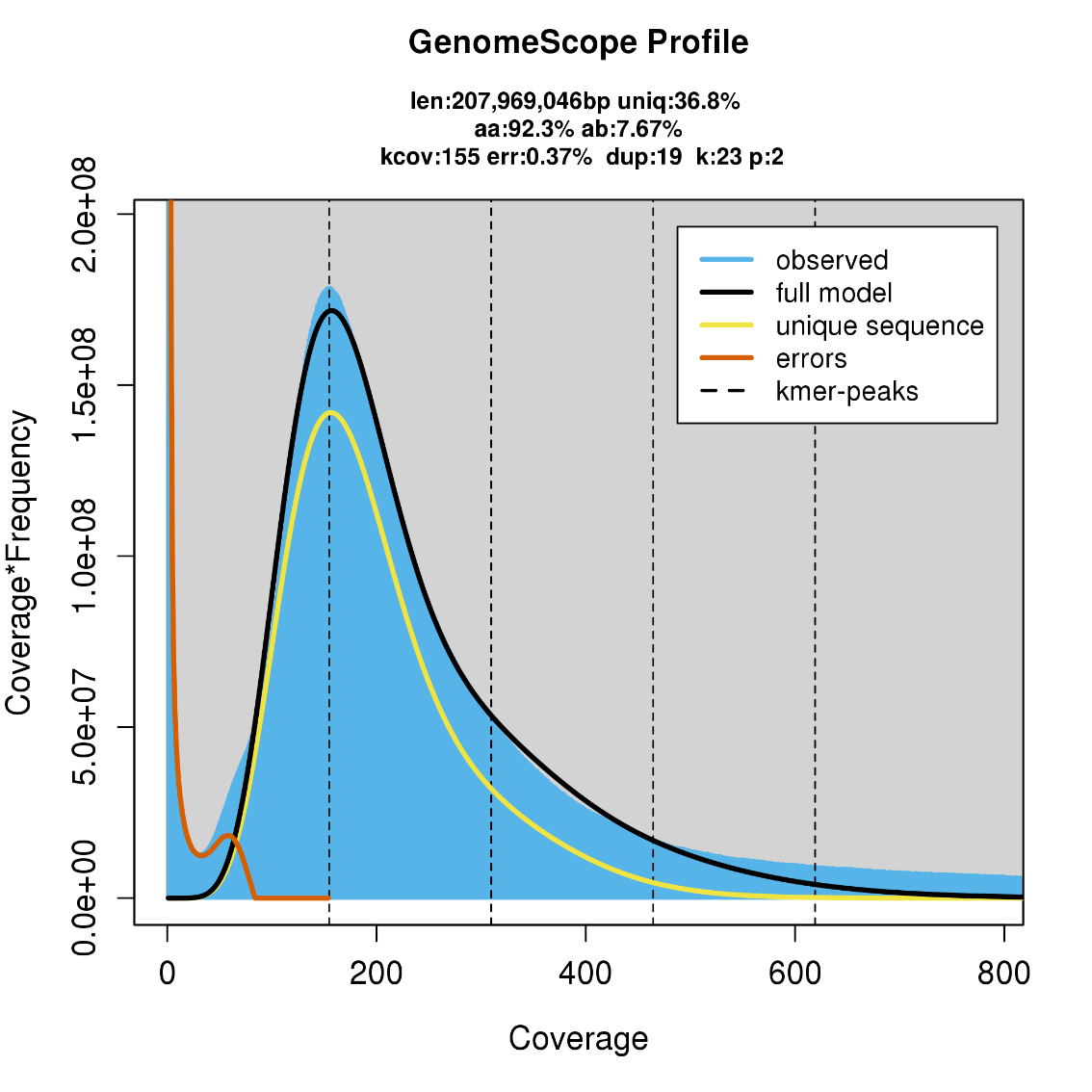


**Figure S1.** The 23-mer analysis of the *P. avium* genome


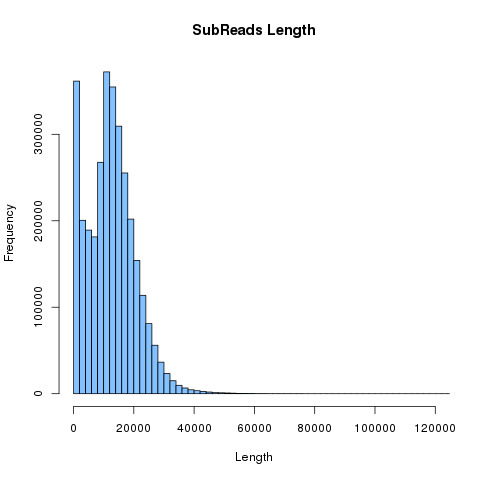


**Figure S2.** Frequency distribution of nanopore sequencing reads.


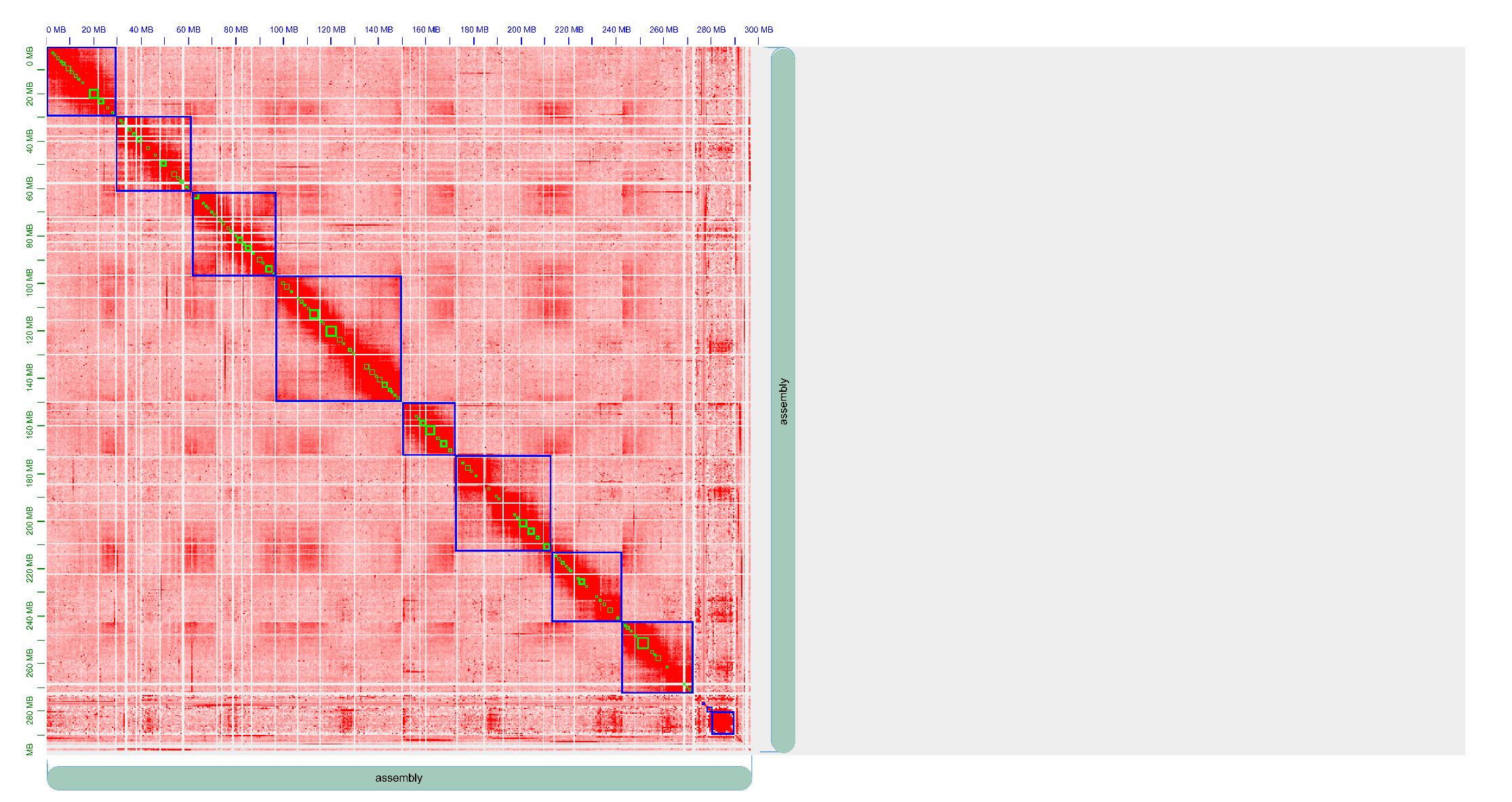


**Figure S3.** High-resolution Hi-C interaction heatmap of *P. avium* genome.


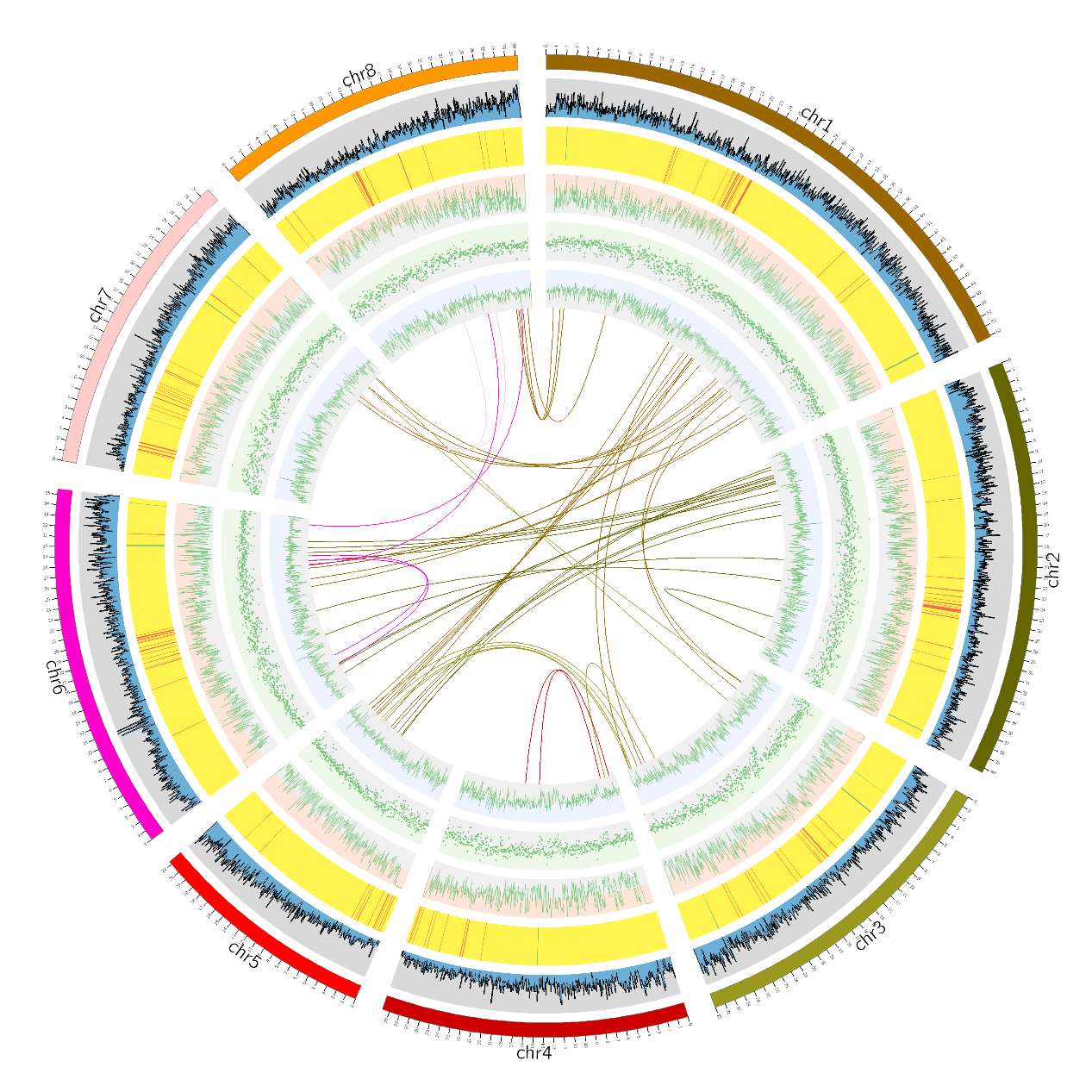


a

b

c

d

e

**Figure S4.** Summary of genome assembly and sequencing analysis of *P. avium*. **(a)** 8 chromosomes (chr. 1–8), **(b)** gene density, **(c)** GC content, and **(d)** Repeat sequence content in 50 kb sliding windows, and **(e)** synteny blocks among *P. avium* chromosomes.

f

e

a

d)

c

b


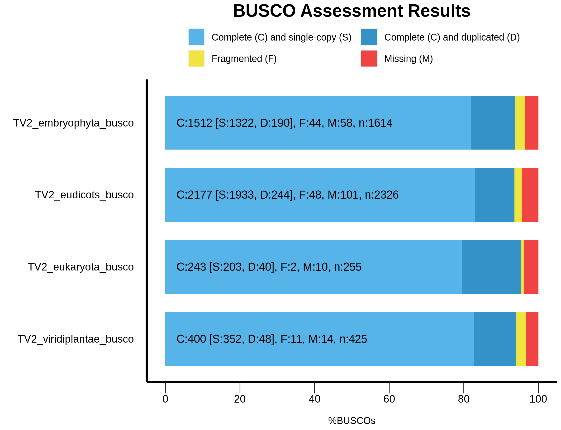

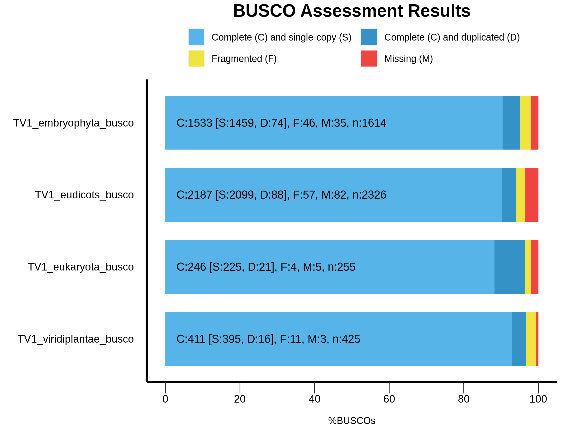

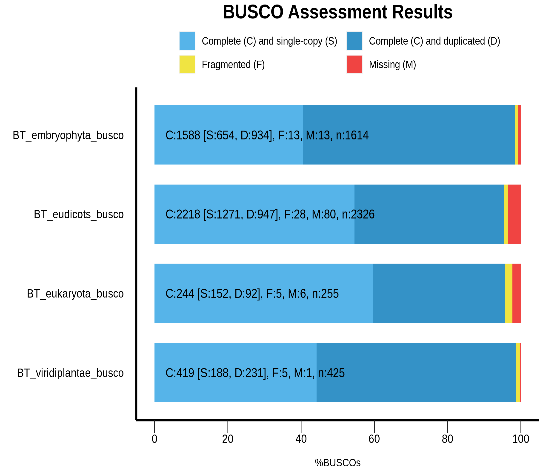

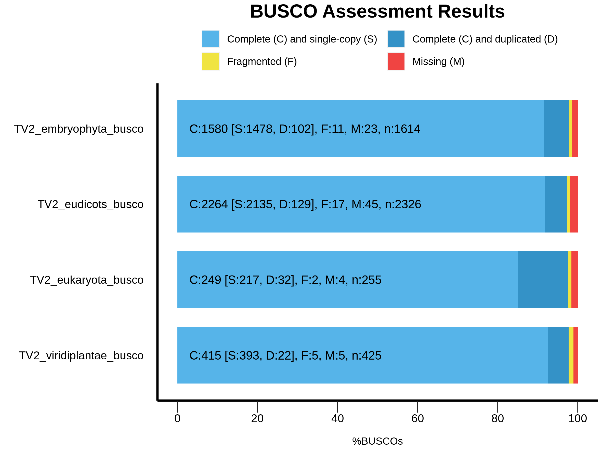

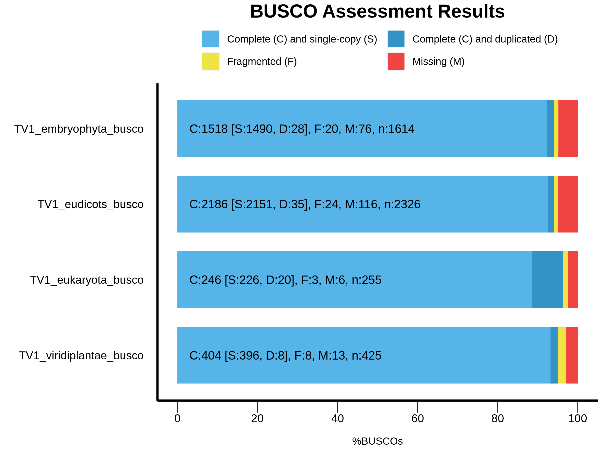

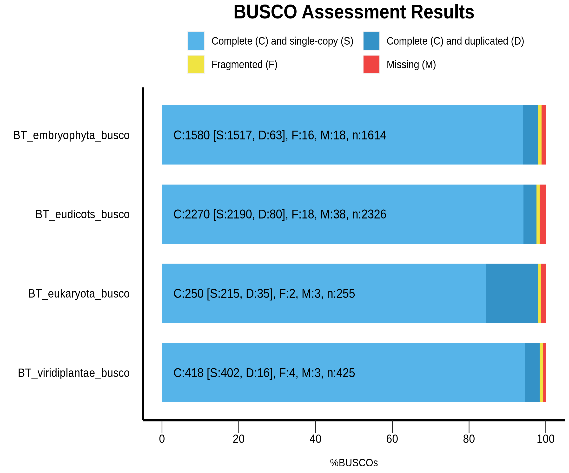


**Figure S5.** Comparison of BUSCO assessment of assembly and annotation genes quality for European sweet cherry; The vertical axis represents the various BUSCO databases (embryophyta, eudicots, eukaryote, viridiplantae), while the horizontal axis represents the number of gene annotations; (a-b) Burlat cherry assembly and annotation; (c-d) Tieton-v2 cherry; (e-f) Tieton-v1 cherry.

**
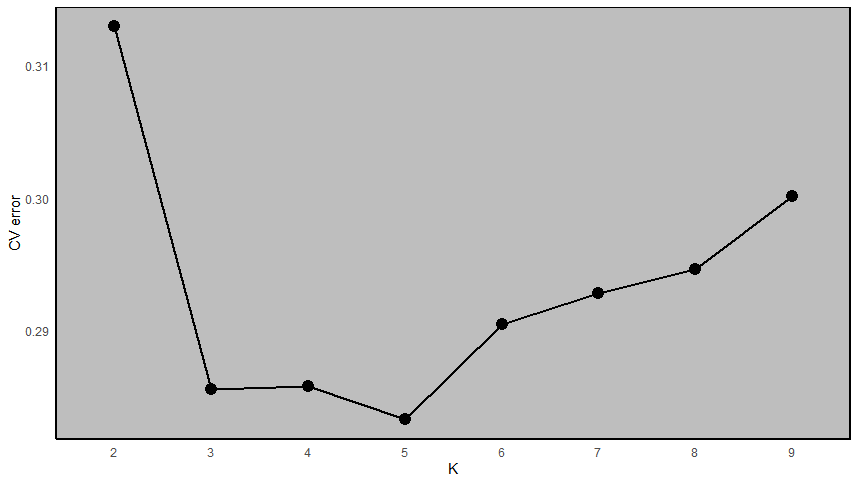
**

**Figure S6.** Cross-validation errors. The x-axis represents the K value, while y-axis indicates the cross-validation errors. The dot shows K = 6 with the lowest cross-validation errors.


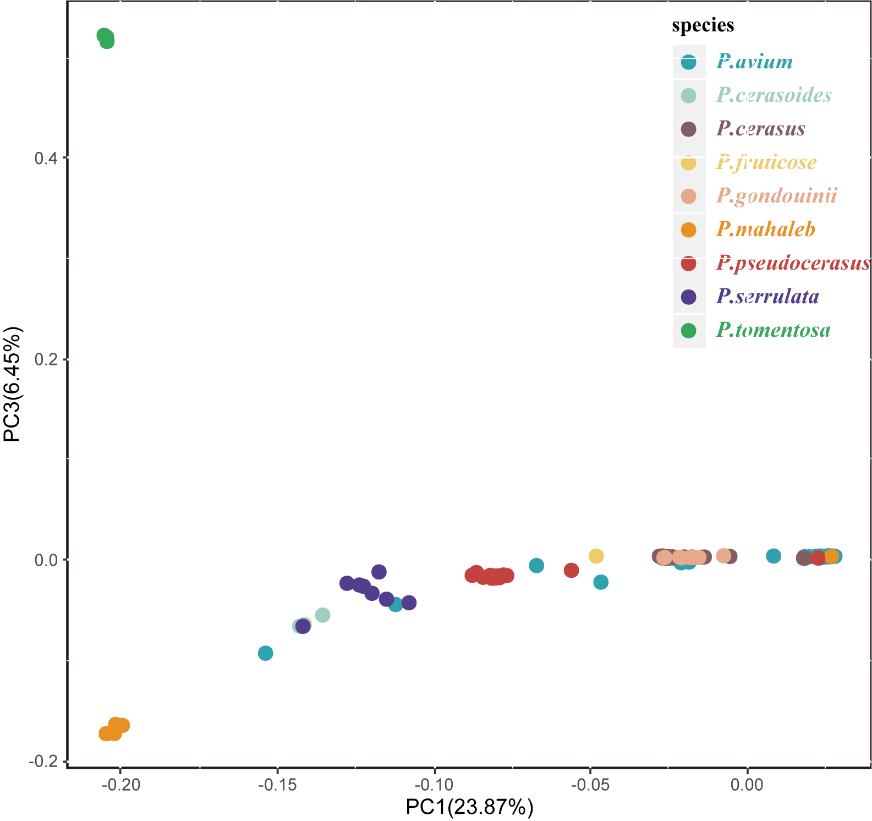


**Fig S7.** Principal component analysis of the first three components of the 384 accessions.

**
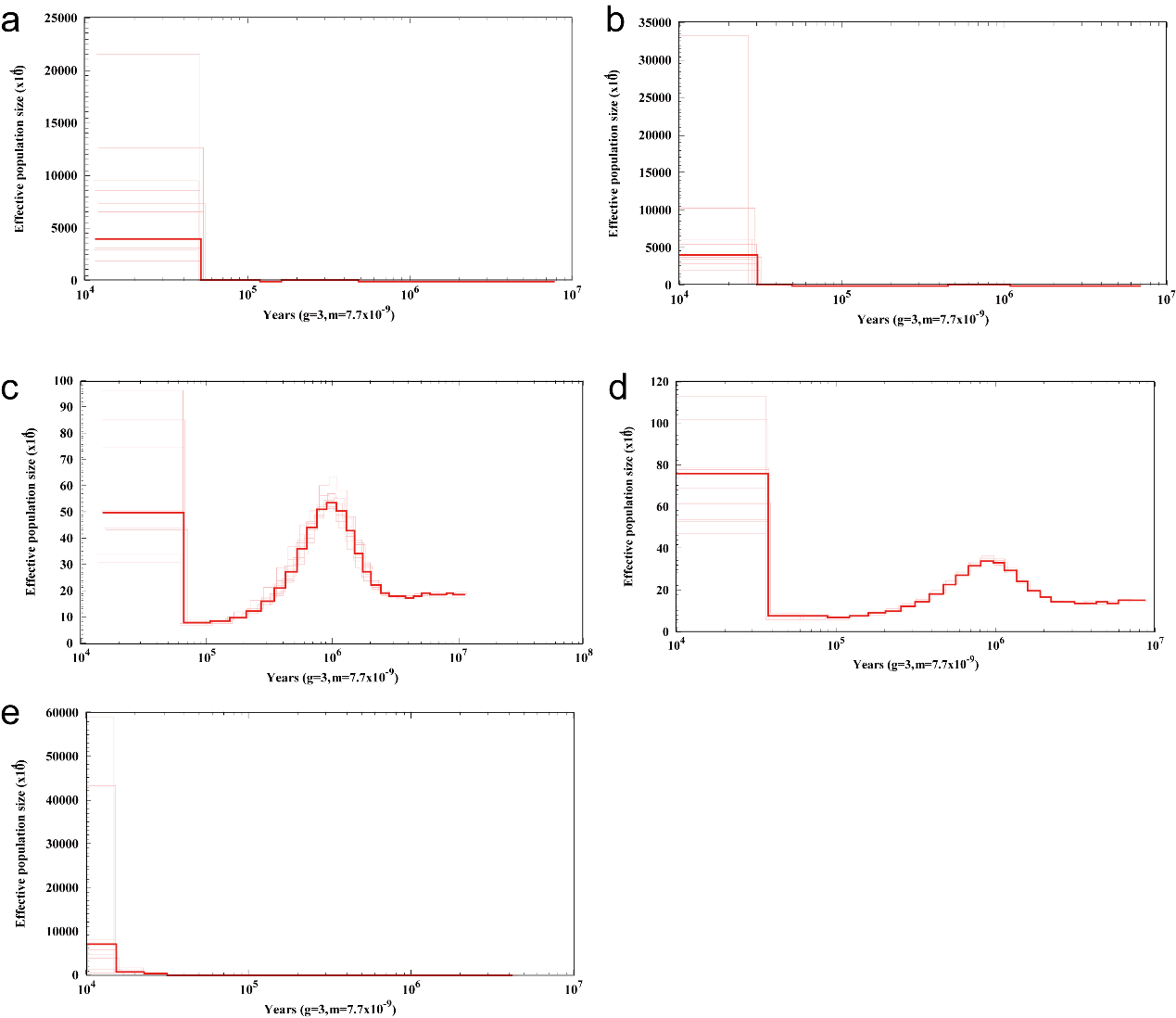
**

**Figure S8.** Demographic history of *Cerasus* (a: *P. pseudocerasus*: b: *P*× *gondouinii*; c: *P. fruticose*, d: *P. cerasus* and e: *P. cerasoides*) germplasm inferred from the estimation of the historical effective population size Ne using the PSMC method.


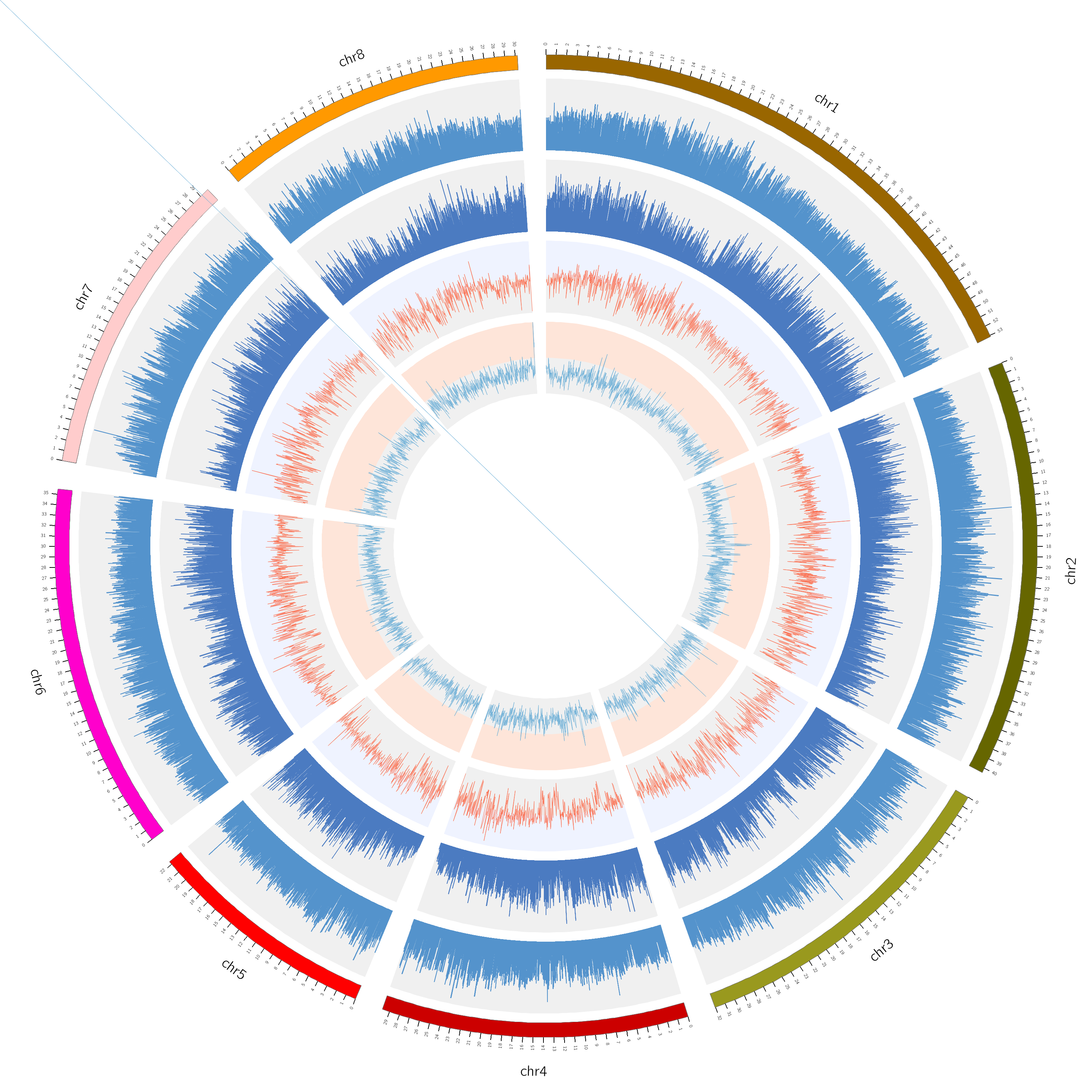


b

c

d

a

e

**Supplementary Figure 9**

**Figure S9.**  Diagram of SNPs found by resequencing of 141 individuals. Circles represent from outermost to innermost, **(a)** 8 chromosomes (chr. 1–8) denoted by different colors, **(b)** SNP abundance bars, **(c)** INDEL abundance bars, **(d)** nucleotide diversity abundance bars, **(e)** Tajama’D abundance bars

(a)


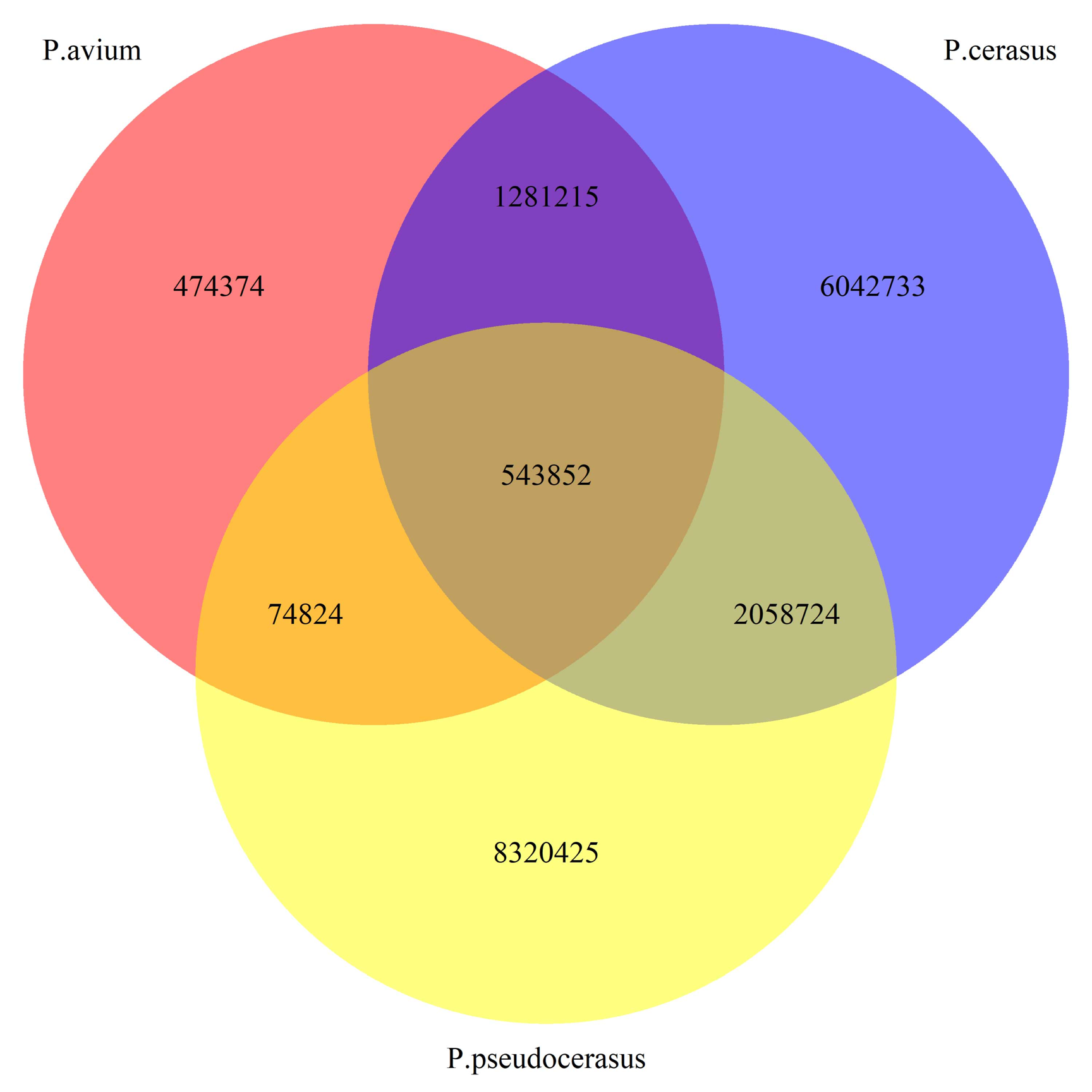

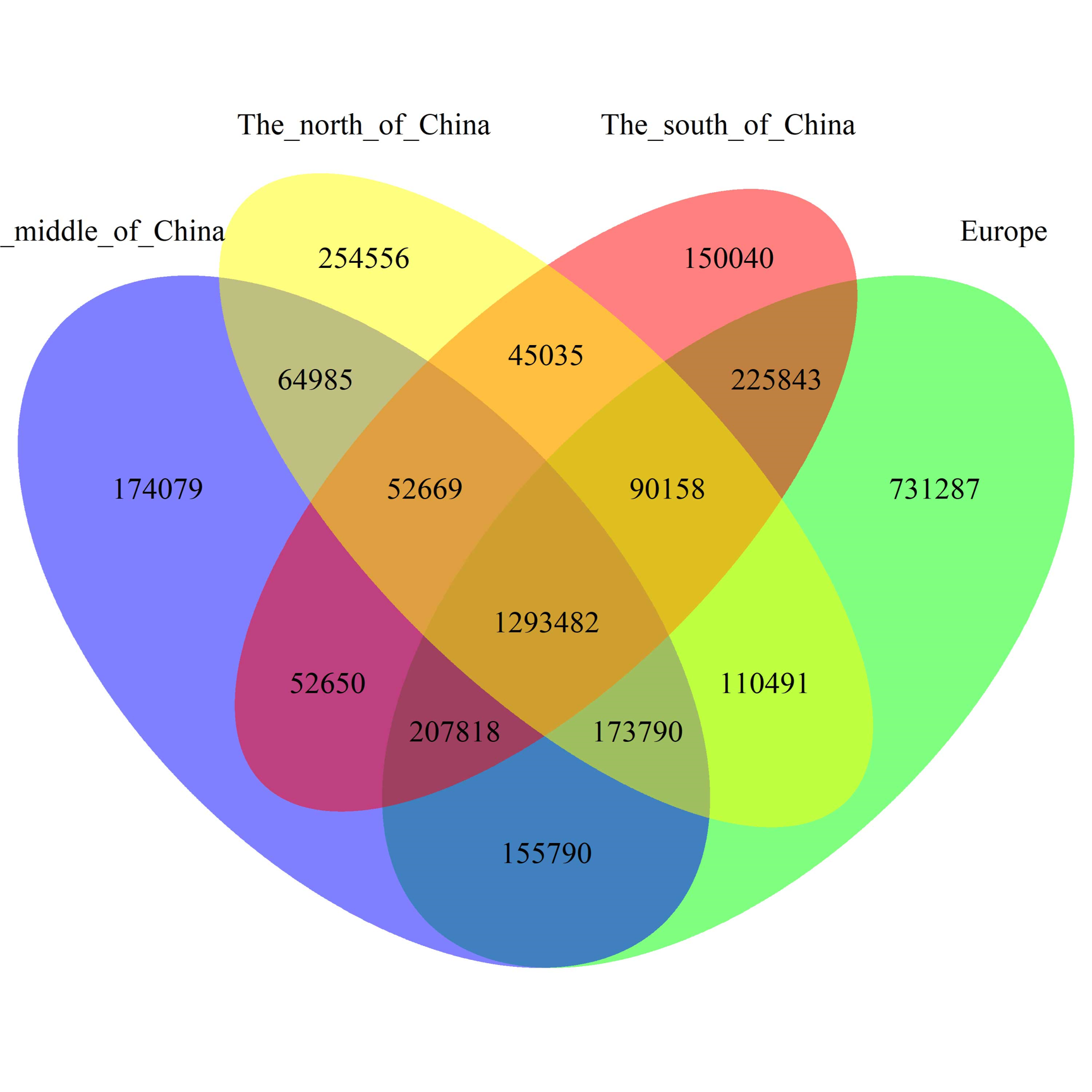


(b)

**Figure S10.** Venn diagrams for SNP variants detected (a) in *P. avium*, *P. cerasus* and *P. pseudocerasus*. (b) among different geographic regions.


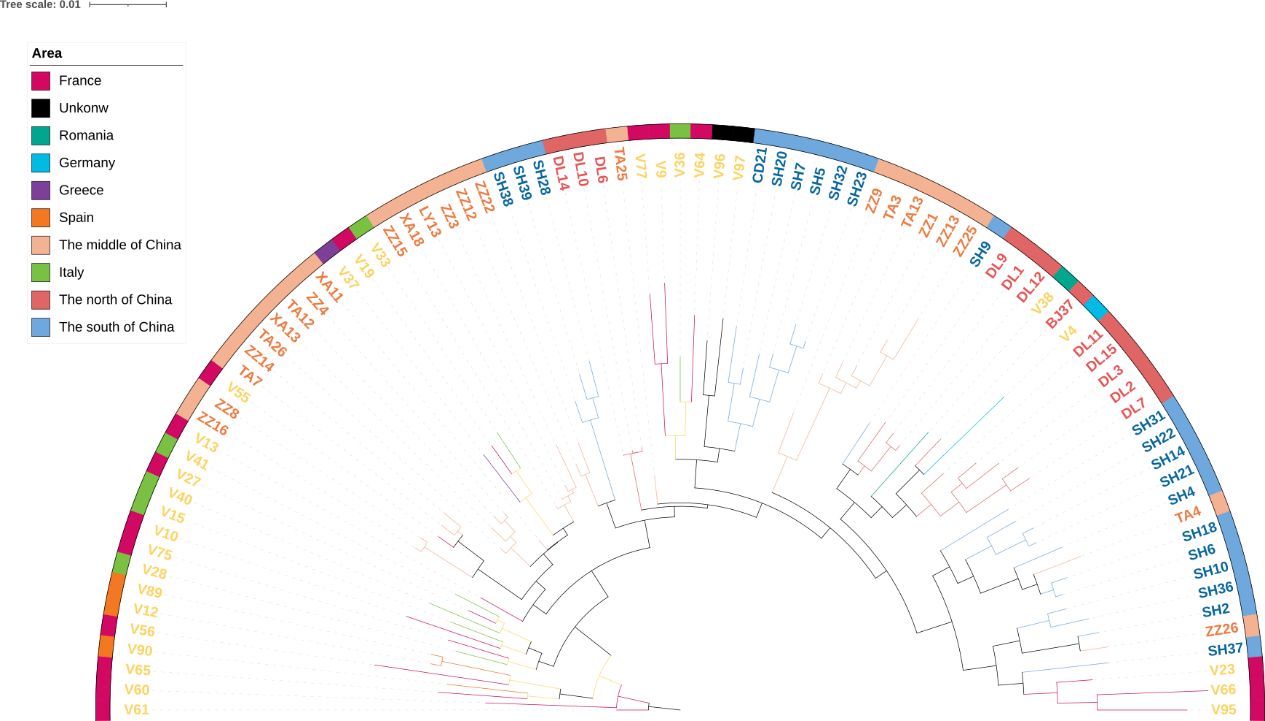


**Figure S11.** Phylogenetic tree of 87 *P. avium* accessions from different Eurasian geographic regions.


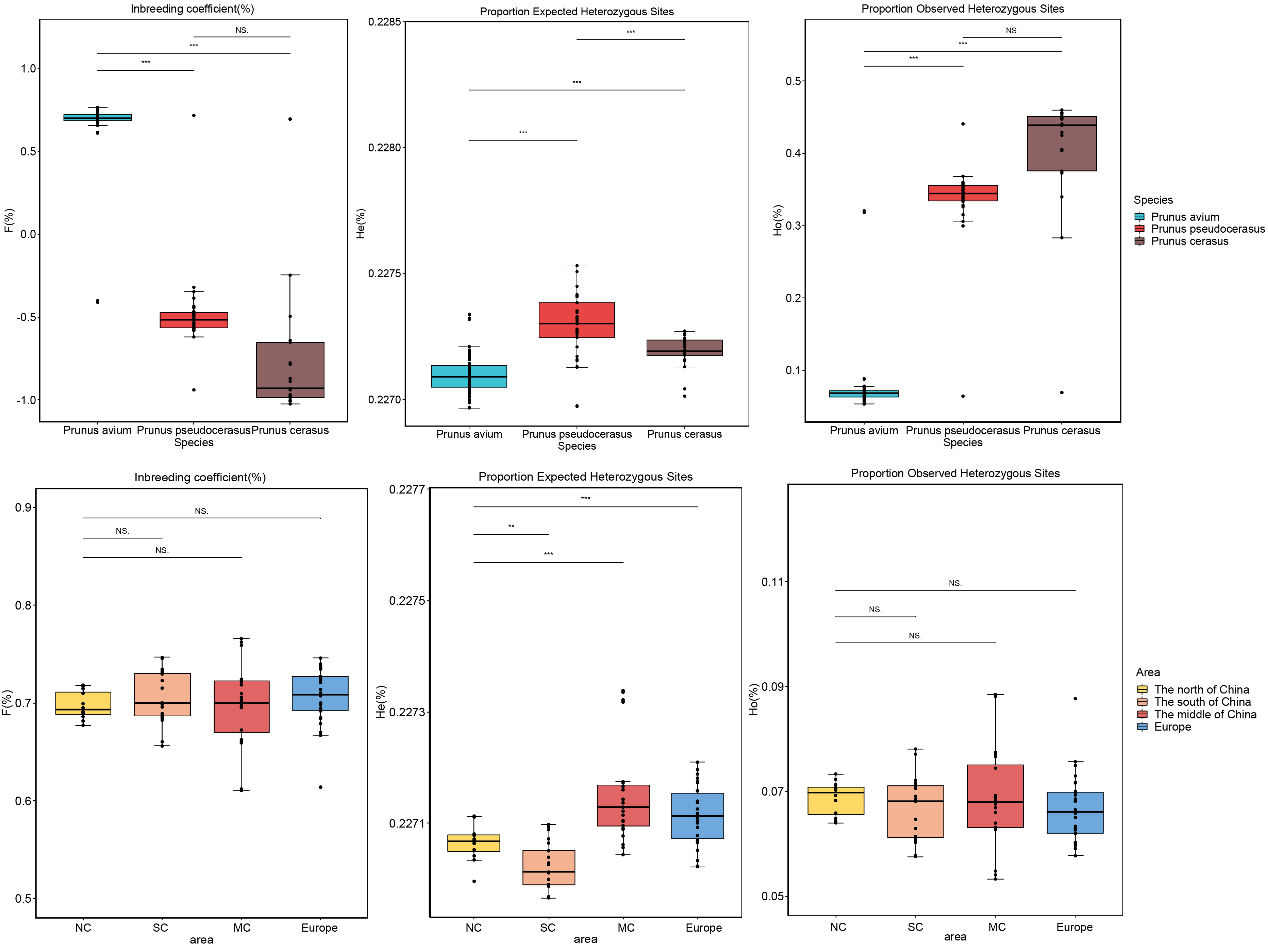


(a)

(b)

**Figure S12.** Inbreeding coefficient, Proportion Observed Heterozygous Sites (%) and Proportion Expected Heterozygous Sites (%) estimation (a)in *P. avium*, *P. cerasus*, and *P. pseudocerasus* species; (b) among different geographic regions. Significances of difference between groups were derived with one-sided t-test. Among them, 0.01 < *p < 0.05; 0.001 < **p < 0.01; ***p < 0.001.

**
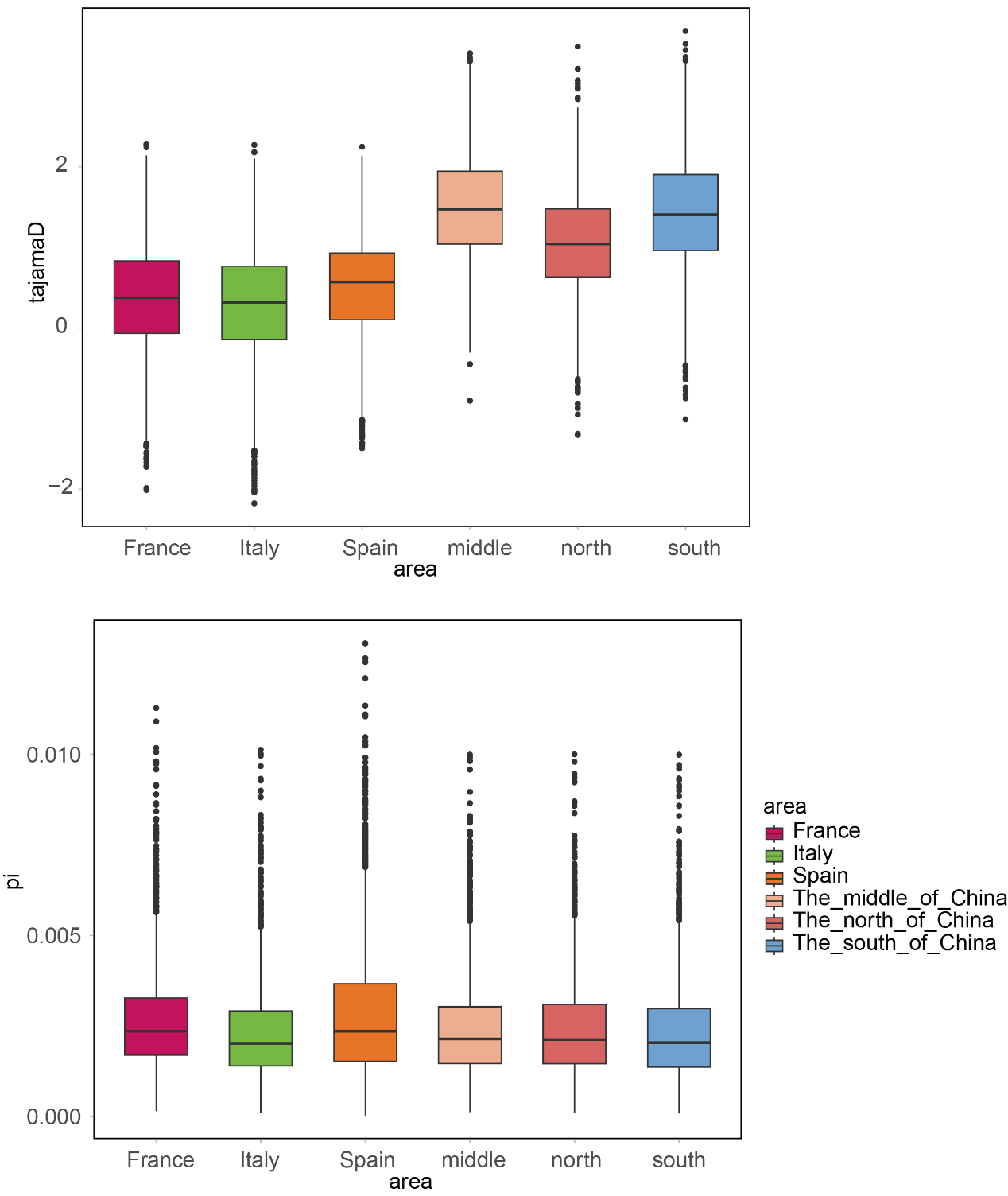
**

(b)

(a)

**Figure S13.** Nucleotide diversity (π) and tajamaD estimation from different geographic regions.


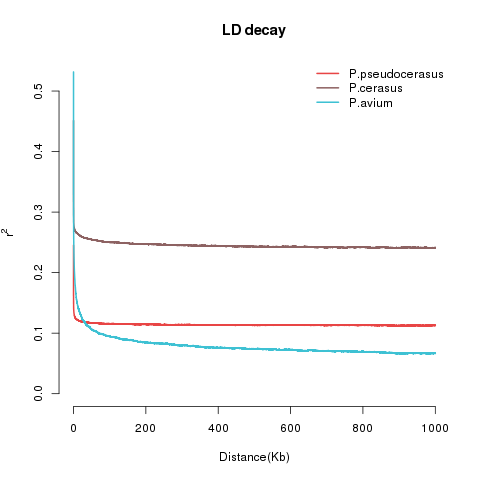
**Figure S14.** Linkage disequilibrium (LD) decay among edible cherries.


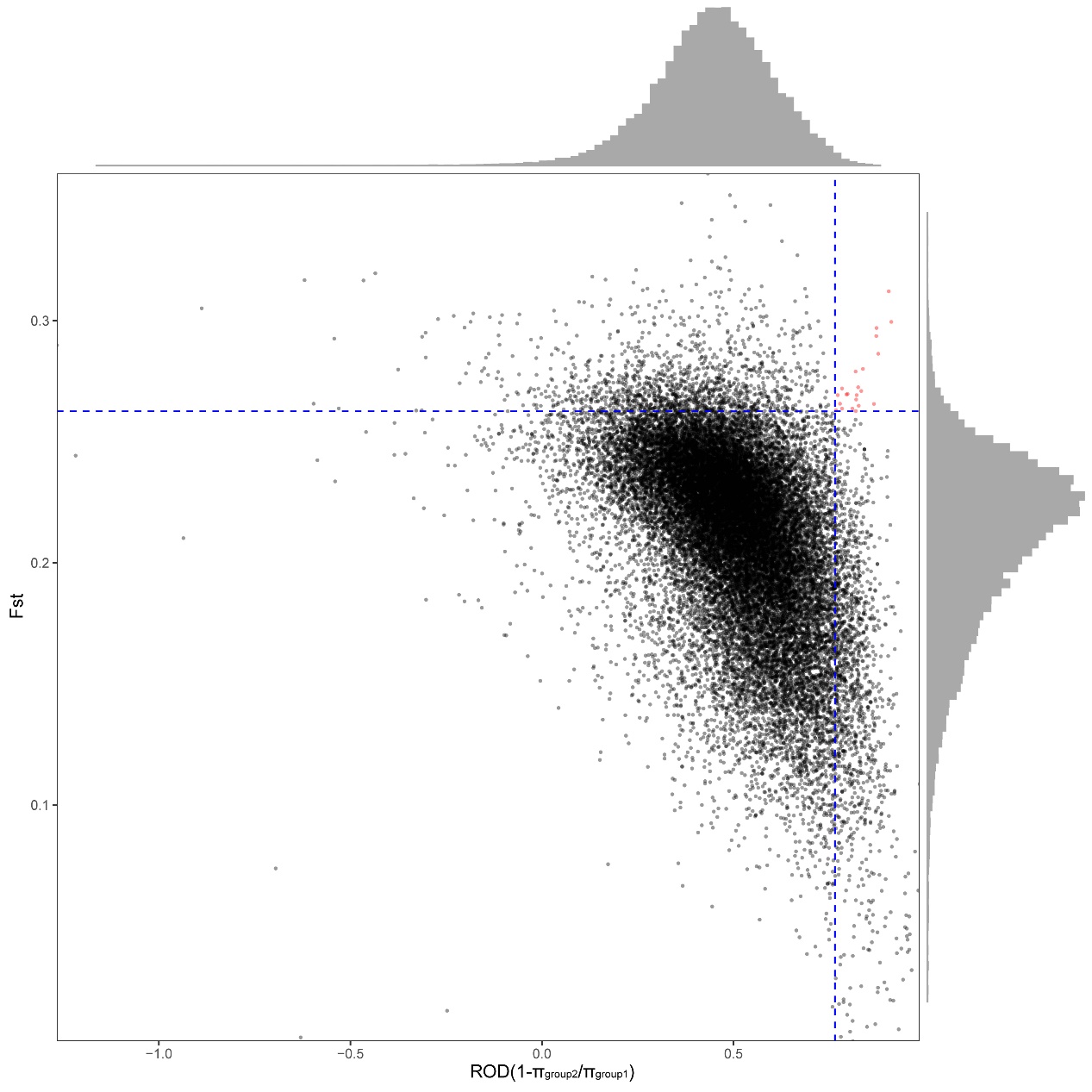


**Figure S15. Selective sweep regions during evolution of *Cerasus* inferred from *F_ST_* and *ROD* statistics of group1 and group2.**

**
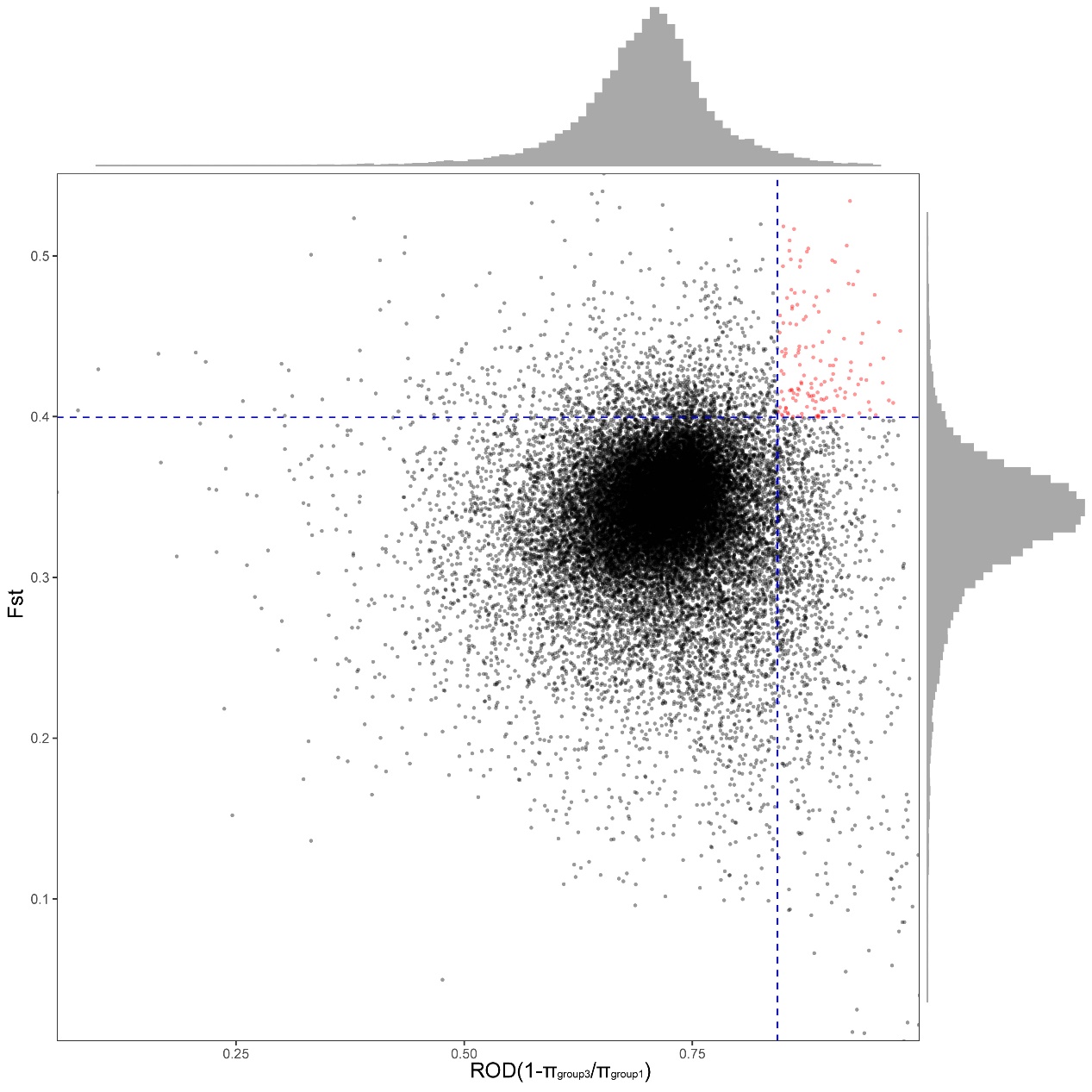
**

**Figure S16. Selective sweep regions during evolution of *Cerasus* inferred from *F_ST_* and *ROD* statistics of group1 and group3.**

**
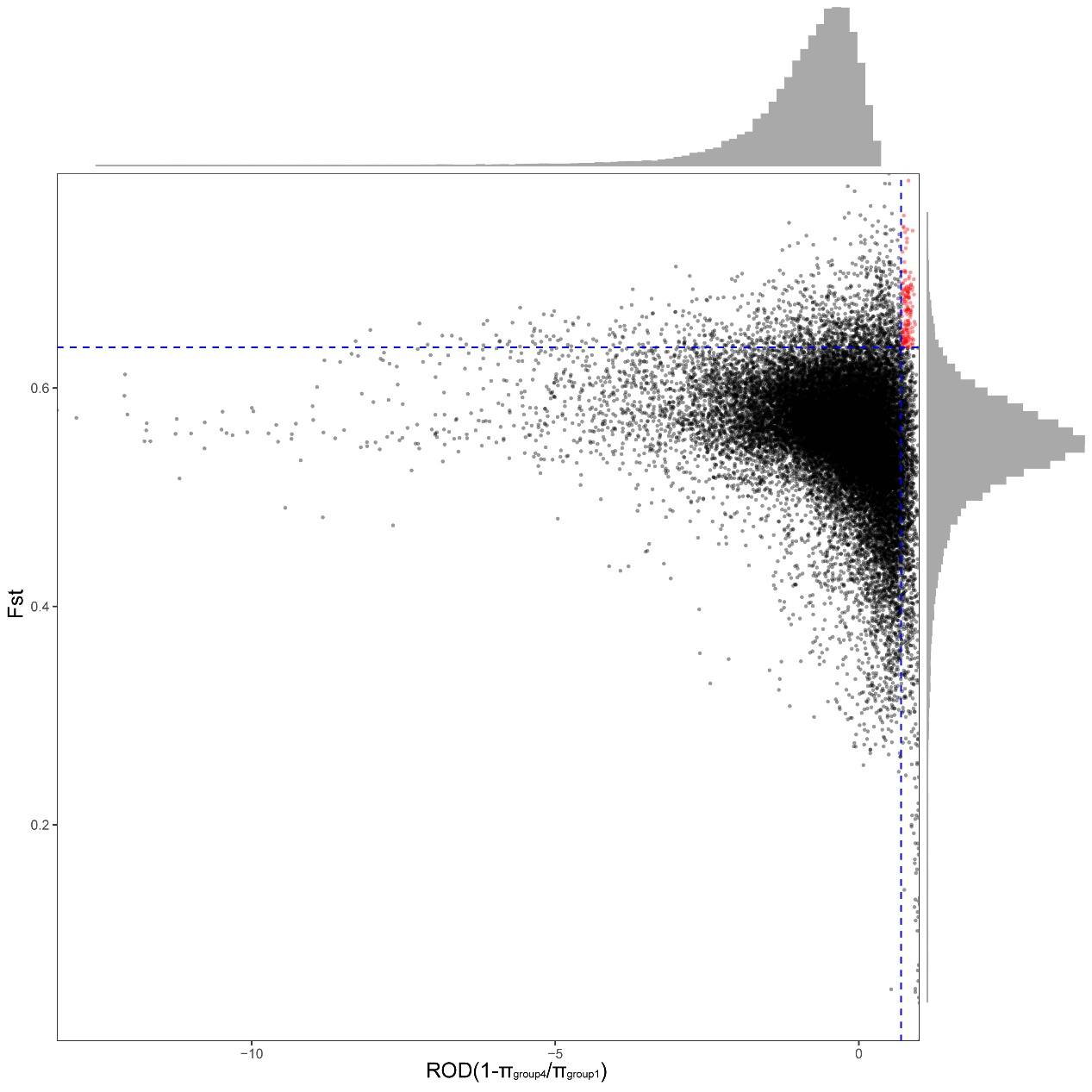
**

**Figure S17. Selective sweep regions during evolution of *Cerasus* inferred from *F_ST_* and *ROD* statistics of group1 and group4.**

**
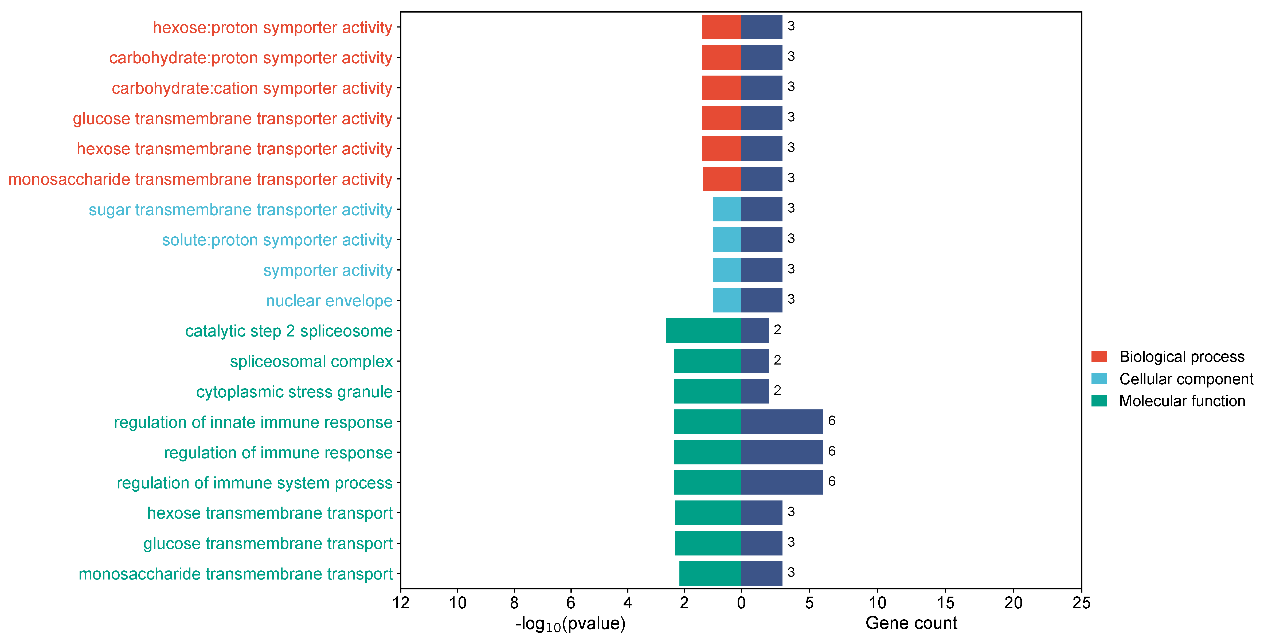
Figure S18.** The enrichment analysis of GO in *P. cerasus*.

**
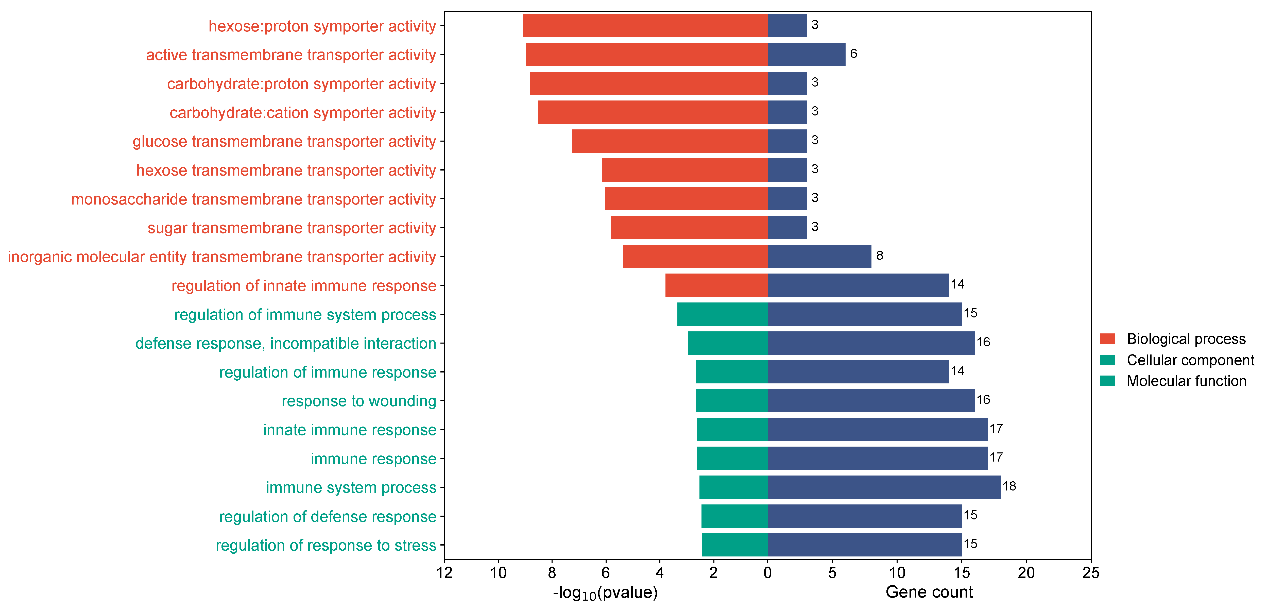
**

**Figure S19.** The enrichment analysis of GO in *P. pseudocerasus*.

**
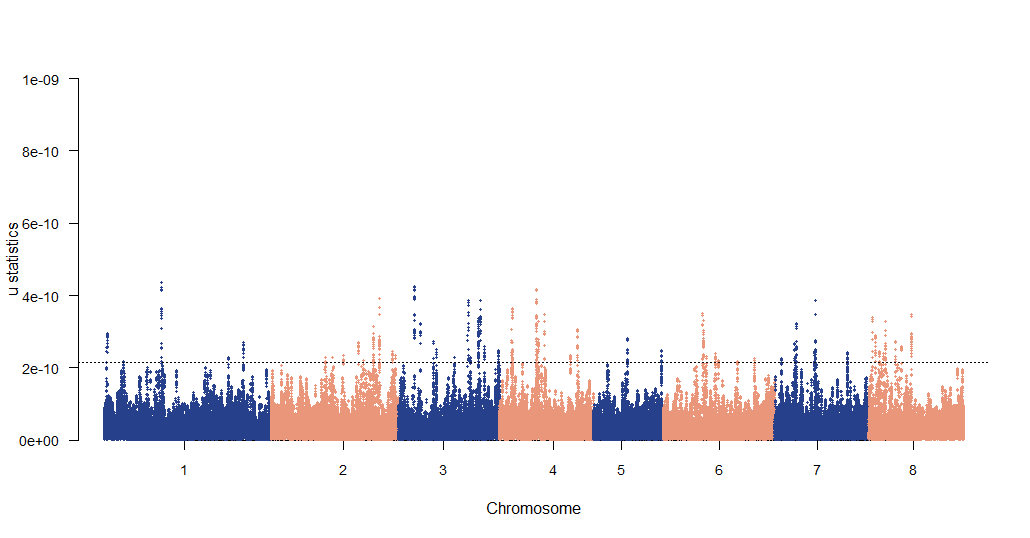
**

**Figure S20.** μ statistics calculated by raisd across the genome in *P. avium*. The dashed lines mark the regions at the top 0.5%.


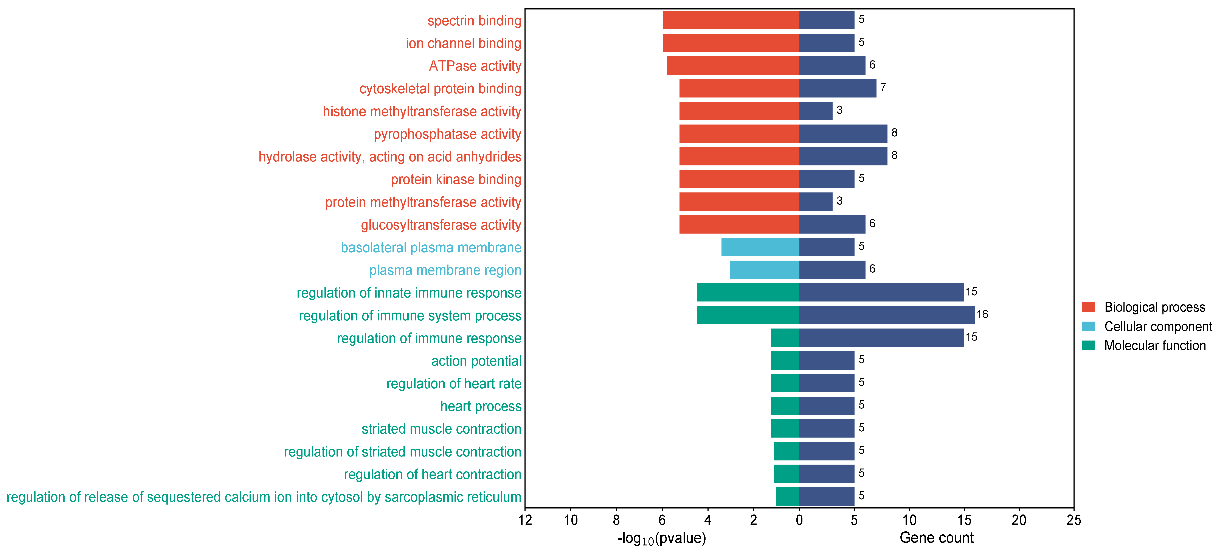


Figure S21. The enrichment analysis of GO in *P. avium*.
